# Mitochondria targeting molecular transporters: synthesis, lipophilic effect, and ionic complex

**DOI:** 10.1080/10717544.2021.2023696

**Published:** 2022-01-11

**Authors:** Akula S. N. Murthy, Sanket Das, Tejinder Singh, Tae-Wan Kim, Nasim Sepay, Seob Jeon, Jungkyun Im

**Affiliations:** a Department of Electronic Materials and Devices Engineering, Soonchunhyang University, Asan, South Korea; b Department of Chemistry, Pohang University of Science and Technology, Pohang, South Korea; c Department of Medical Life Science, Soonchunhyang University, Asan, South Korea; d Department of Obstetrics and Gynecology, College of Medicine, Soonchunhyang University Cheonan Hospital, Cheonan, South Korea; e Department of Chemical Engineering, Soonchunhyang University, Asan, South Korea

**Keywords:** Mitochondria, targeted-delivery, drug delivery, molecular transporter, mitochondrial dysfunction

## Abstract

As mitochondria are potential therapeutic targeting sites for the treatment of human diseases, delivering cytotoxic drugs, antioxidants, and imaging molecules to mitochondria can provide new therapeutic opportunities. In an attempt to develop a new mitochondria-targeting vector, we synthesized sorbitol-based molecular transporters with multiple guanidines, measured their partition coefficients, compared their targeting efficiency using fluorescent images and Pearson's correlation coefficients, and studied cellular uptake mechanisms. To increase the targeting ability of these molecular transporters to mitochondria, alanine-naphthalene as a lipophilic group was attached to the molecular transporter, which improved translocation across cellular membranes and led to higher accumulation in mitochondria. The molecular transporter was able to form an ionic complex with antibiotics, resulting in low cell viability. These data demonstrate that the molecular transporter with a lipophilic group could be utilized as a potential drug delivery vector for treating mitochondrial dysfunction.

## 1. Introduction

Mitochondria generate ATP by oxidative phosphorylation and are thus regarded as cell powerhouses. They also engage in a wide range of cellular processes including homeostasis of calcium concentration, regulation of cellular redox states, and metabolism of carbohydrates, fatty acids, and amino acids (Wallace et al., 2010; Murphy et al., 2016). Additionally, mitochondria are involved in the modulation of cellular apoptosis (Wang & Youle, 2009). However, pathological events and the free radicals generated by the mitochondrial respiratory system can damage mitochondria. Genetic mutation of mitochondrial DNA or nuclear DNA can also create defects in mitochondrial components (Smith et al., 2012). Since mitochondria are linked to several biological processes, any such negative features in mitochondria can lead to mitochondrial dysfunction, causing other diverse human diseases including cancers, type 2 diabetes, obesity, ischemia-reperfusion injury, and neurodegenerative diseases, such as Alzheimer’s disease, Parkinson’s disease, and Huntington’s disease (Lin & Beal, 2006; Prime et al., 2009; Lu et al., 2016; Intihar et al., 2019).

Therefore, the mitochondrion is a specific organelle that can serve as a potential therapeutic target for drugs in various diseases. We expect drugs targeting mitochondria to inhibit mitochondrial damage and resolve abnormal mitochondrial functions. Thereby, several human diseases require such therapeutic interventions even in diseases not originally caused by mitochondrial damage. One mitochondria-targeting therapies is antioxidants treatment to decrease the oxidative damage caused by reactive oxygen species (ROS) (Hoye et al., 2008). Since the mitochondrion is a major site that produces ROS by oxidative phosphorylation, mitochondria-targeted antioxidants can effectively minimize mitochondrial dysfunction and improve related diseases (Battogtokh et al., 2018). Furthermore, delivering cytotoxic drugs and imaging molecules to mitochondria can provide multiple therapeutic opportunities.

Some mitochondria-targeting strategies have been developed in an effort to deliver drugs to mitochondria. They include lipophilic cations, mitochondria-penetrating peptides, and short peptides or oligomers containing guanidines (Fernández-Carneado et al., 2005; Ozawa et al., 2007; Yousif et al., 2009; Szeto & Schiller, 2011; Jean et al., 2014; Kubi et al., 2018). However, lipophilic cations are not effective for delivering large cargoes, and they could have intrinsic toxicity (Murphy & Smith, 2000; Lu et al., 2016). Mitochondria-penetrating peptides are highly prone to hydrolysis by proteases in blood plasma. The toxicity of peptide vehicles having guanidines or cations should be considered as well as their ability to conjugate with drugs. In addition, cell-penetrating peptides, such as HIV-TAT and transportan (which have good cellular internalization and thus attracted interest in preclinical studies) have failed to achieve efficient organelle targeting (Al-Taei et al., 2006; Song et al., 2019). Thus, it is challenging to develop a novel vehicle that can both target mitochondria and deliver drugs that are cell-membrane impermeable.

Previously, our research group reported several kinds of molecular transporters that showed efficient cellular uptake and high affinity toward mitochondria (Maiti et al., 2007; Jin et al., 2011; Lee et al., 2017). The molecular transporters were non-peptides, and they had carbohydrates as scaffolds to which multiple guanidines were attached *via* linkers. When drugs like paclitaxel, camptothecin, AZT, 5-Fu, and ibuprofen were covalently conjugated to the molecular transporters, the conjugates were well-internalized into the cells (Maiti et al., 2007; Im et al., 2011; Jeong et al., 2018). Some of the drug-conjugated molecular transporters demonstrated dominant cellular localization in mitochondria. However, the selectivity of these molecular transporters for mitochondria has not been studied in-depth.

**Figure 1.**
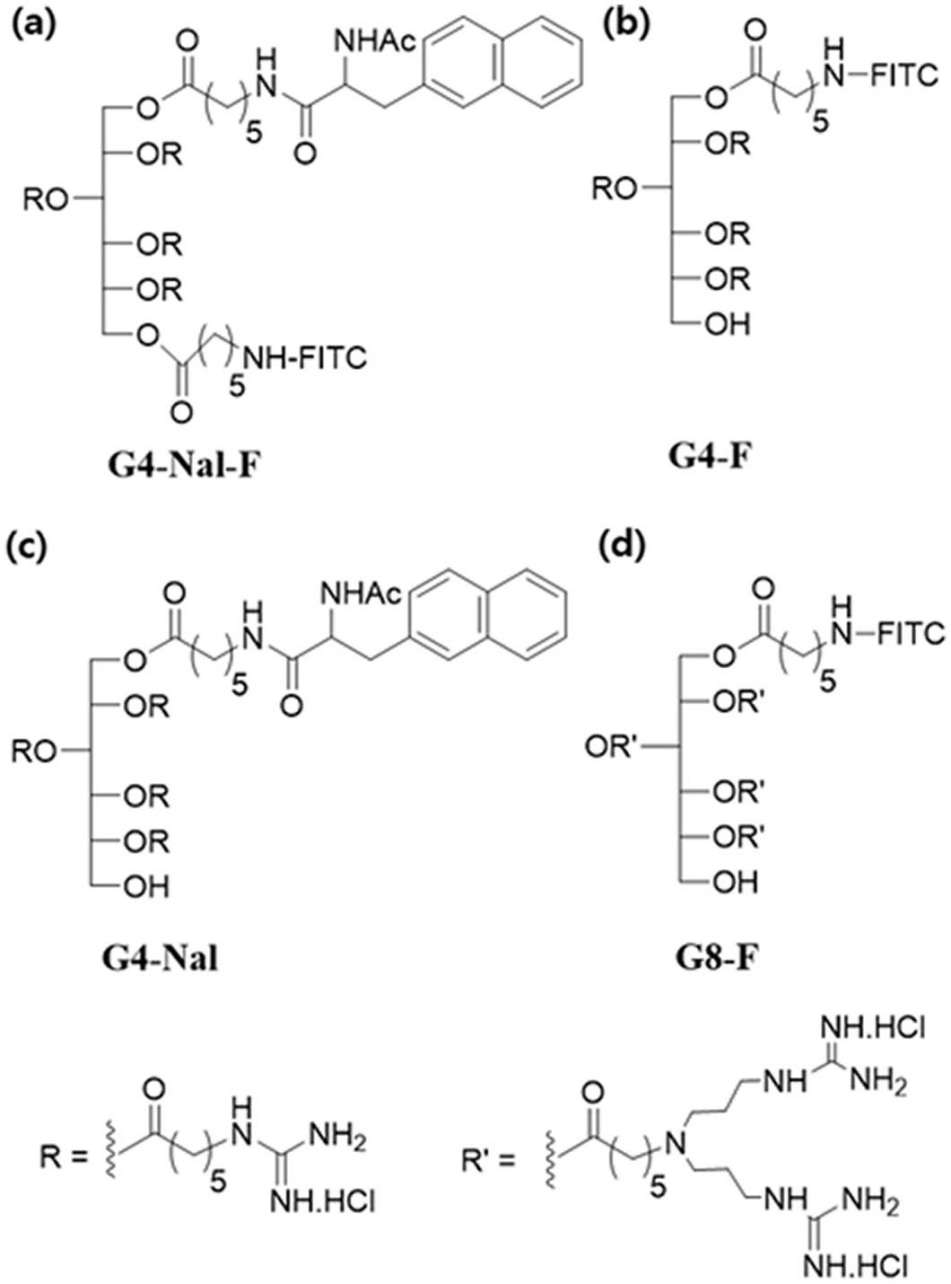
The chemical structure of the sorbitol-based molecular transporters synthesized in this study. (a) **G4-Nal-F**, (b) **G4-F**, (c) **G4-Nal**, and (d) **G8-F**.

In this research, we synthesized a sorbitol-based molecular transporter (**G4-Nal-F**) linked with four guanidines, a fluorescence probe, and a lipophilic group (Figure 1(a)) to enhance the ability of the molecular transporter to target mitochondria. The mitochondrial proton pumps present in the inner mitochondrial membrane generate membrane potential that creates a gradient concentration of protons for ATP production. The mitochondrial membrane potential (*ΔΨ*_m_) is −180 mV, and the matrix is more negative because protons are moving from the matrix into the intermembrane space of the mitochondria (Smith et al., 2011). This is the driving force for the transportation of cationic molecules across the inner mitochondrial membrane, and the accumulation of cations into the mitochondrial matrix can be predicted using the Nernst equation (Azzone et al., 1984; Murphy & Smith, 2000).

$$\text{Membrane potential }(\mathrm{mV})=61.5\;\log\{{\left[\mathrm{cation}\right]}_{\mathrm{in}}/{\left[\mathrm{cation}\right]}_{\mathrm{out}}\}\;(\mathrm{at}\;37{}^{\circ}C)$$

Thus, a 100- to 500-fold increase of mitochondrial uptake can be expected for each guanidinium. Furthermore, the membrane potential of mitochondria is much larger than other organelles within cells, and this will act as a strong attractive force for molecular transporters having guanidines to accumulate predominantly in mitochondria. Moreover, guanidines are essential for translocation over cellular membranes, as studied before (Im et al., 2013). Additionally, an alanine-naphthalene was selected as the lipophilic group and was tethered to the molecular transporter. Previous publications reported that lipophilic cations, such as triphenylphosphonium can transport across the hydrophobic bilayer of membranes since the hydrophobic groups contribute to the delocalization of the phosphocation during translocation (Murphy, 2008; Smith et al., 2011; Madak & Neamati, 2015). For the same reason, we expected the attached lipophilic group to help the molecular transporter pass through the lipid bilayer more easily and accumulate in the mitochondria. We compared the mitochondria-targeting capability of these new amphiphilic molecular transporters based on the partition coefficient (Log *P*) and the number of guanidines, studied the cellular uptake mechanism, investigated the delivery of antibiotics to mitochondria, and examined cytotoxicity.

## 2. Experimental

### 2.1. Materials and methods

α-D-Glucose, tritylchloride (TrCl), triethylamine (Et_3_N), trimethylsilyl chloride (TMSCl), pyridine, 1-ethyl-3-(3-dimethylaminopropyl) carbodiimide (EDC), 4-dimethylaminopyridine (DMAP), tetra-*n*-butylammonium fluoride (TBAF), succinic anhydride, diisopropylethylamine (DIPEA), fluorescein-5-isothiocyanate (FITC), trifluoroacetic acid (TFA), 1-octanol, 3-(4,5-dimethylthiazol-2-yl)-2,5-diphenyltetrazolium bromide (MTT), imipramine hydrochloride, chlorpromazine hydrochloride, and methyl-β-cyclodextrin were purchased from Sigma-Aldrich Korea (Yongin, Korea). 17-AHA-geldanamycin was supplied by Focus Biomolecules (PA, USA). Geldanamycin (**GA**), 3-(2-naphthyl)-DL-alanine, and sodium borohydride (NaBH_4_) were purchased from Alfa Aesar (Korea Fisher Science Co., Ltd.). Ammonium acetate (NH_4_OAc) and acetic anhydride (Ac_2_O) were purchased from Samchun Chemicals (Seoul, Korea). Dulbecco's phosphate-buffered saline (DPBS, pH 7.4), Roswell Park Memorial Institute (RPMI) 1640, high glucose Dulbecco's modified Eagle's medium (DMEM), fetal bovine serum (FBS), and trypsin/EDTA were obtained from Gibco™, Thermo Fisher Scientific (Loughborough, UK). MitoTracker™ Red CMXRos, lysotracker Red DND-99, Hoechst 33342, and rhodamine B-dextran (MW 10,000) were purchased from Thermo Fisher Scientific (Invitrogen, OR, USA). Milli‐Q purified water (18.2 MΩ) was used to prepare all the aqueous solutions. Medium pressure liquid chromatography (MPLC) was performed on CombiFlash RF + Lumen instrument with an integrated UV and ELS detector (MA, USA). Analytical reverse phase-high performance liquid chromatography (RP-HPLC) and preparative RP-HPLC were performed on an Agilent 1220 Infinity LC Chemstation (Santa Clara, CA, USA) with Eclipse XDB-C18 (reverse phase) and C18 monochromatic column, respectively. Nuclear magnetic resonance (NMR) spectra data were recorded with Bruker ASPECT 300 or Jeol Resonance JNM-ECS400 instruments. For analysis of high molecular weight compounds, matrix-assisted laser desorption/ionization (MALDI-TOF) data were obtained from BIONEER (Daejon, Korea).

### 2.2. Characterization of synthetic compounds

#### 2.2.1. 1-O-Trityl-D-sorbitol (2)

Compound **2** was prepared according to the previous literature procedure (Maiti et al., 2007). First, α-D-glucose was dissolved in dry pyridine at rt, and Et_3_N was added dropwise to the solution under an inert atmosphere. To the solution mixture, TrCl was added portionwise at rt. A clear solution was observed after 24 h of reaction time. The reaction mixture was diluted with CH_2_Cl_2_ and washed with 1 N HCl(aq) solution. The organic layer was dried over Na_2_SO_4_, filtered, and concentrated. The crude product was purified by column chromatography (using 70–230 mesh size silica with CombiFlash RF + Lumen instrument with an integrated UV detector (MA, USA); 10% MeOH in CH_2_Cl_2_ as mobile phase) to give compound 1-*O*-trityl-α-D-glucose. Next, 1-*O*-trityl-α-D-glucose was dissolved in dry MeOH at rt, and the solution was allowed to cool to 0 °C. To the solution, NaBH_4_ was charged portionwise under an inert atmosphere. After 16 h, the starting material was reduced to compound **2**. The crude product was directly used in the next step without further purification. The analytical data were exactly matched to the data mentioned in the reference article.

#### 2.2.2. 1,2,3,4,5-O-(trimethylsilyl)-6-O-trityl-D-sorbitol (3)

Compound **2** (1.4 g, 3.26 mmol) was dissolved in dry pyridine (20 mL) and allowed to cool to 0 °C. To the solution, TMSCl (8.3 mL, 65.2 mmol) was added dropwise over a period of 30 min under cold conditions. After the addition of TMSCl, the reaction temperature was slowly raised to rt and stirred for 3 days under an inert atmosphere. After completion of the reaction (monitored by TLC), the white solid precipitate of the reaction mixture was dissolved in cold water and extracted with ether. The organic layer was separated, dried over Na_2_SO_4_, filtered, and concentrated. The crude product was purified by column chromatography (230–400 mesh size silica gel, 1% EtOAc in hexane as mobile phase) to get the desired compound **3** (1.1 g, 43%) as colorless liquid. ^1^H NMR (δ, CDCl_3_): −0.04 (s, 9H), −0.01 (s, 9H), 0.04 (s, 9H), 0.09 (s, 9H), 0.19 (s, 9H), 3.08–3.24 (m, 3H), 3.38–3.49 (m, 2H), 3.64–3.78 (m, 3H), 4.25–4.27 (m, 1H), 7.18–7.29 (m, 9H), 7.45 (dd, *J* = 6.9, 1.2 Hz, 6H).

#### 2.2.3. 1-O-Trityl-2,3,4,5-tetra-O-(trimethylsilyl)-D-sorbitol (4)

NH_4_OAc (431 mg, 5.6 mmol) was added portionwise over a period of 2 h into a solution of compound **3** (1.1 g, 1.4 mmol) in MeOH/CH_2_Cl_2_ mixture (6:1). The reaction was forced to stop after the consumption of half of the starting materials. The reaction mixture was diluted with CH_2_Cl_2_ and washed with 50% brine solution, dried over Na_2_SO_4_, filtered, and concentrated. The crude product was purified by column chromatography (230–400 mesh size silica gel, 10% EtOAc in hexane as mobile phase) to get the desired compound **4** (470 mg, 47%) as colorless sticky liquid. ^1^H NMR (δ, CDCl_3_): −0.02 (s, 9H), 0.03 (s, 9H), 0.04 (s, 9H), 0.19 (s, 9H), 2.25 (t, *J* = 5.5 Hz, 1H), 3.06 (AB_q_, *J* = 7.0, 3.0 Hz, 1H), 3.30 (t, *J* = 8.0 Hz, 1H), 3.50 (t, *J* = 6.5 Hz, 1H), 3.56–3.82 (m, 4H), 4.23 (d, *J* = 5.5 Hz, 1H), 7.19–7.28 (m, 9H), 7.45 (d, *J* = 7.5 Hz, 6H).

#### 2.2.4. 1-O-(N-Cbz-6-aminohexanoyl)-2,3,4,5-tetra-O-(trimethylsilyl)-6-O-trityl-D-sorbitol (5)

Compound **4** (550 mg, 0.77 mmol), *N*-Cbz protected aminohexanoic acid (410 mg, 1.54 mmol), EDC (443 mg, 2.31 mmol), and DMAP (28 mg, 0.23 mmol) were dissolved in dry CH_2_Cl_2_ (10 mL), and the solution was stirred under inert atmosphere. After 12 h, the reaction mixture was diluted with water and washed with 5% NaHCO_3_(aq) solution. The organic layer was dried over Na_2_SO_4_, filtered, and concentrated in a vacuum to give the crude product, which was purified by column chromatography (using 70–230 mesh size silica; 10% EtOAc in hexane as mobile phase) to give compound **5** (600 mg, 81%) as foamy white solid. ^1^H NMR (δ, CDCl_3_): −0.04 (s, 9H), 0.01 (s, 9H), 0.03 (s, 9H), 0.17 (s, 9H), 1.32–1.64 (m, 6H), 2.27 (t, *J* = 7.5 Hz, 2H), 3.12 (AB_q_, *J* = 3.8, 2.5 Hz, 1H), 3.16–3.20 (m, 2H), 3.28 (t, *J* = 7.5 Hz, 1H), 3.46 (t, *J* = 5.5 Hz, 1H), 3.71 (dd, *J* = 6.0, 1.5 Hz, 1H), 3.90–3.93 (m, 1H), 4.00 (dd, *J* = 11, 7.5 Hz, 1H), 4.15 (d, *J* = 6.5 Hz, 1H), 4.27 (dd, *J* = 11.5, 2 Hz, 1H), 4.71 (brs, 1H), 5.08 (s, 2H), 7.20–7.45 (m, 20H).

#### 2.2.5. 1-O-(N-Cbz-6-aminohexanoyl)-6-O-trityl-D-sorbitol (6)

Compound **5** (600 mg, 0.62 mmol) was dissolved in dry THF (10 mL) and cooled to 0 °C. TBAF (3.1 mL, 3.1 mmol) was added dropwise over a period of 15 min under cold conditions and stirred at 0 °C for another 45 min. After complete deprotection, the reaction mixture was diluted with CH_2_Cl_2_. The organic layer was washed with 50% brine solution, dried over Na_2_SO_4_, and concentrated. The crude product was purified by column chromatography (230–400 mesh size silica gel, 3% MeOH in CH_2_Cl_2_ as mobile phase). Compound **6** (330 mg, 79%) was obtained as foamy white solid. ^1^H NMR (δ, CDCl_3_): 1.30–1.67 (m, 6H), 2.30 (t, *J* = 7 Hz, 2H), 3.02 (brs, 1H), 3.15 (dd, *J* = 12.5, 6.5 Hz, 3H), 3.30–3.43 (m, 4H), 3.74 (brs, 1H), 3.79 (brs, 1H), 3.88 (brs, 1H), 3.98 (brs, 1H), 4.18 (d, *J* = 5.5 Hz, 2H), 4.90 (brs, 2H), 5.05 (s, 2H), 7.22–7.43 (m, 15H).

#### 2.2.6. 1-O-(N-Cbz-6-aminohexanoyl)-2,3,4,5-[N′,N″-bis(tert-butoxycarbonyl-guanidinohexanoyl]-6-O-trityl-D-sorbitol (7)

Compound **6** (150 mg, 0.22 mmol) was dissolved in dry CH_2_Cl_2_ (4 mL), and the solution was transferred to a vial containing freshly dried 4 Å molecular sieves powder. To the solution were added **Compound A** (*N′*,*N″*-bis-*tert*-butoxycarbonyl-guanidino hexanoic acid, 500 mg, 1.32 mmol), EDC (295 mg, 1.54 mmol), DMAP (53 mg, 0.44 mmol), and dry CH_2_Cl_2_ (4 mL) at rt. After stirring for 3 days, the reaction mixture was filtered, and the filtrate was washed with saturated NaHCO_3_(aq) solution. The organic layer was dried over Na_2_SO_4_, filtered, and concentrated in vacuum to give the crude product, which was purified by column chromatography (using 70–230 mesh size silica, 35% EtOAc in hexane as mobile phase) to give compound **7** (300 mg, 65%) as a foamy white solid. ^1^H NMR (δ, CDCl_3_): 1.26–1.58 (m, 102H), 1.93–2.28 (m, 10H), 3.03–3.34 (m, 12H), 4.00–4.08 (m, 1H), 4.36–4.34 (m, 1H), 5.00 (s, 2H), 5.01–5.03 (m, 1H), 5.13–5.21 (m, 2H), 5.41–5.45 (m, 2H), 7.14–7.32 (m, 20H), 8.22 (brs, 4H), 11.47 (brs, 4H); ^13 ^C NMR (δ, CDCl_3_): 172.59, 172.37, 172.09, 171.88, 163.57, 163.56, 156.35, 156.04, 153.26, 153.22, 143.19, 136.78, 128.64, 128.37, 127.90, 127.88, 127.79, 127.14, 86.98, 82.93, 82.88, 79.04, 79.01, 69.42, 69.31, 68.71, 68.29, 61.72, 61.60, 40.80, 40.64, 40.62, 33.84, 33.73, 33.67, 33.65, 29.55, 28.76, 28.71, 28.26, 28.01, 26.36, 26.27, 26.24, 26.14, 24.37, 24.31, 24.24, 24.17.

#### 2.2.7. 1-O-(6-Aminohexanoyl)-2,3,4,5-[N′,N″-bis(tert-butoxycarbonyl-guanidinohexanoyl]-6-O-trityl-D-sorbitol (8)

Compound **7** (300 mg, 0.14 mmol), and 10% Pd/C (100 mg) were dissolved in dry CH_2_Cl_2_ (0.5 mL) and MeOH (5 mL). After hydrogenolysis under 30 psi of H_2_(g) for 6 h, the reaction mixture was filtered through celite, washed with 50% MeOH in CH_2_Cl_2_, and concentrated. Product **8** (270 mg, 0.13 mmol) was directly used in the next step without further purification.

#### 2.2.8. 1-O-6-[2-Acetamido-3-(naphthalen-2-yl)propanaminohexanoyl]-2,3,4,5-[N′,N″-bis(tert-butoxycarbonyl-guanidinohexanoyl]-6-O-trityl-D-sorbitol (9)

Compound **8** (136 mg, 0.07 mmol), **Nal** [(2-acetamido-3-(naphthalen-2-yl)-propanoicacid), 36 mg, 0.14 mmol], EDC (40 mg, 0.21 mmol), and DMAP (3 mg, 0.021 mmol) were dissolved in dry CH_2_Cl_2_ (5 mL), and the solution was stirred for 24 h at rt under inert atmosphere. After 24 h, the reaction mixture was diluted with CH_2_Cl_2_ and washed with saturated NaHCO_3_(aq) solution. The organic layer was dried over Na_2_SO_4_, filtered, and concentrated in vacuum to give the crude product, which was purified by column chromatography (using 70–230 mesh size silica, 40% EtOAc in hexane as mobile phase) to give compound **9** (100 mg, 65%) as foamy white solid. ^1^H NMR (δ, CDCl_3_): 1.23–1.61 (m, 102H), 1.88 (s, 3H), 1.92–2.32 (m, 10H), 2.97–3.22 (m, 6H), 3.31–3.39 (m, 8H), 3.99–4.05 (m, 1H), 4.30–4.34 (m, 1H), 4.60–4.66 (m, 1H), 5.06–5.07 (m, 1H), 5.20–5.29 (m, 1H), 5.42–5.47 (m, 1H), 5.94–6.00 (m, 1H), 6.44–6.46 (m, 1H), 7.28–7.43 (m, 18H), 7.60 (s, 1H), 7.71–7.78 (m, 3H), 8.27 (brs, 4H), 11.48 (brs, 4H); ^13 ^C NMR (δ, CDCl_3_): 172.79, 172.62, 172.58, 172.23, 172.10, 172.09, 170.73, 170.71, 169.90, 163.76, 163.73, 156.24, 153.47, 153.42, 143.38, 134.47, 133.60, 132.53, 128.83, 128.38, 128.08, 127.97, 127.76, 127.66, 127.50, 127.32, 126.36, 125.89, 87.17, 83.17, 83.13, 83.11, 79.31, 79.28, 69.55, 69.51, 68.95, 68.51, 62.08, 61.80, 54.95, 54.92, 40.87, 40.84, 39.35, 39.14, 39.12, 34.04, 33.97, 33.88, 33.86, 33.69, 28.97, 28.92, 28.83, 28.45, 28.20, 26.59, 26.51, 26.47, 26.26, 24.61, 24.54, 24.46, 24.39, 24.29, 24.27, 23.30.

#### 2.2.9. 1-O-6-[2-Acetamido-3-(naphthalen-2-yl)propanaminohexanoyl]-2,3,4,5-[N′,N″-bis(tert-butoxycarbonyl-guanidinohexanoyl]-6-O-D-sorbitol (10)

Compound **9** (30 mg, 0.014 mmol) was dissolved in dry CH_2_Cl_2_ (1 mL) and cooled to −15 °C. 1 mL of 5% TFA in CH_2_Cl_2_ was added dropwise under cold condition and stirred for additional 15 min. The reaction mixture was diluted with CH_2_Cl_2_ and washed with 10% NaHCO_3_(aq) solution. The organic layer was dried over Na_2_SO_4_, filtered, and concentrated in vacuum to give the crude product, which was purified by column chromatography (using 70–230 mesh size silica, 3% MeOH in CH_2_Cl_2_ as mobile phase) to afford compound **10** (24 mg, 90%) as foamy white solid. ^1^H NMR (δ, CDCl_3_): 1.10–1.63 (m, 102H), 1.95 (s, 3H), 2.12–2.40 (m, 10H), 3.02–3.21 (m, 4H), 3.34–3.38 (m, 8H), 3.50–3.59 (m, 1H), 3.69–3.81 (m, 1H), 3.91–4.00 (m, 1H), 4.25–4.29 (m, 1H), 4.63–4.68 (m, 1H), 4.87–4.92 (m, 1H), 5.25–5.30 (m, 1H), 5.42–5.50 (m, 2H), 6.20–6.70 (m, 2H), 7.32–7.78 (m, 7H), 8.20 (brs, 4H), 11.47 (brs, 4H); ^13 ^C NMR (δ, CDCl_3_): 173.35, 173.18, 173.00, 172.93, 172.90, 172.72, 172.51, 172.48, 172.36, 172.29, 171.11, 170.93, 170.48, 170.21, 163.75, 156.29, 153.49, 134.48, 133.66, 132.60, 128.43, 128.36, 128.20, 128.17, 127.86, 127.82, 127.73, 127.58, 127.55, 126.37, 126.31, 126.91, 125.86, 83.20, 79.39, 71.04, 70.92, 69.75, 69.61, 69.28, 69.09, 68.85, 68.72, 62.14, 62.08, 60.23, 60.10, 55.02, 54.89, 40.91, 39.25, 39.19, 39.14, 38.71, 34.10, 34.05, 33.99, 33.83, 33.80, 32.08, 28.95, 28.49, 28.26, 26.63, 26.53, 26.49, 26.11, 26.07, 24.69, 24.66, 24.61, 24.45, 24.43, 24.36, 24.21, 23.34, 23.27.

#### 2.2.10. 1-O-6-[2-Acetamido-3-(naphthalen-2-yl)propanaminohexanoyl]-2,3,4,5-[N′,N″-bis(tert butoxycarbonyl-guanidinohexanoyl]-6-O-(N-Cbz-6-aminohexanoyl)-D-sorbitol (11)

Compound **10** (20 mg, 0.01 mmol), *N*-Cbz protected aminohexanoic acid (5 mg, 0.02 mmol), EDC (6 mg, 0.03 mmol), and DMAP (1 mg, 0.008 mmol) were dissolved in dry CH_2_Cl_2_ (3 mL), and the solution was stirred under inert atmosphere for 2 days. After completion, the reaction mixture was diluted with water and washed with 5% NaHCO_3_(aq) solution. The organic layer was dried over Na_2_SO_4_, filtered, and concentrated in vacuum to give the crude product, which was purified by column chromatography (using 70–230 mesh size silica, 2% MeOH in CH_2_Cl_2_ as mobile phase) to give compound **11** (20 mg, 90%) as foamy white solid. ^1^H NMR (δ, CDCl_3_): 1.30–1.59 (m, 108H), 1.94 (s, 3H), 2.11–2.36 (m, 12H), 3.02–3.22 (m, 6H), 3.37–3.38 (m, 8H), 3.95–4.06 (m, 2H), 4.22–4.34 (m, 2H), 4.63–4.65 (m, 1H), 5.00–5.21 (m, 5H), 5.38 (brs, 2H), 6.02 (brs, 1H), 6.42 (brs, 1H), 7.25–7.42 (m, 8H), 7.61 (s, 1H), 7.73–7.78 (m, 3H), 8.28 (brs, 4H), 11.49 (brs, 4H); ^13 ^C NMR (δ, CDCl_3_): 173.04, 172.86, 172.69, 172.47, 172.35, 172.15, 170.84, 170.00, 163.77, 156.65, 156.28, 153.48, 136.93, 134.55, 133.66, 132.59, 128.64, 128.42, 128.18, 128.14, 128.02, 127.82, 127.72, 127.57, 126.39, 125.92, 83.21, 79.39, 69.36, 68.77, 68.61, 68.40, 66.66, 61.92, 61.44, 54.98, 40.92, 39.40, 39.09, 34.01, 33.99, 33.91, 33.74, 29.86, 29.76, 29.06, 28.96, 28.49, 28.26, 26.63, 26.57, 26.54, 26.50, 26.35, 26.29, 24.66, 24.58, 24.50, 24.38, 24.32, 23.34.

#### 2.2.11. 1-O-6-[2-Acetamido-3-(naphthalen-2-yl)propanaminohexanoyl]-2,3,4,5-[N′,N″-bis(tert-butoxycarbonyl-guanidinohexanoyl]-6-O-(6-aminohexanoyl)-D-sorbitol (12)

Compound **11** (35 mg, 0.016 mmol), and 10% Pd/C (15 mg) were dissolved in CH_2_Cl_2_ (0.5 mL) and MeOH (5 mL). The reaction mixture was stirred under 30 psi of H_2_(g) for hydrogenolysis. After 24 h, the reaction mixture was filtered through celite, washed with 50% MeOH in CH_2_Cl_2_, and concentrated. The product **12** (35 mg, 0.016 mmol) was directly used in the next step without further purification.

#### 2.2.12. 1-O-6-[2-Acetamido-3-(naphthalen-2-yl)propanaminohexanoyl]-2,3,4,5-(6-guanidinohexanoyl)-6-O-[6-(fluoresceinyl-5-thioureido)-hexanoyl]-D-sorbitol.4HCl (G4-Nal-F)

Compound **12** (35 mg, 0.016 mmol), Et_3_N (6 μL, 0.04 mmol), and FITC (8 mg, 0.02 mmol) were dissolved in 2 mL of dry THF/EtOH mixture (2:4) and the reaction mixture was stirred at rt in dark under inert atmosphere. After 16 h, the reaction mixture was concentrated, and the crude product was dissolved in 10 mL CH_2_Cl_2_. The organic layer was washed with water, dried over Na_2_SO_4_, and filtered. The filtrate was concentrated, dried in a vacuum, and the resulting solid (42 mg) was dissolved in 1 M HCl(g) in EtOAc solution (2 mL) and stirred at rt. After 24 h, yellow precipitation was observed. The reaction mixture was filtered, and the residue was washed several times with EtOAc, THF, and CH_2_Cl_2_. The solid was dissolved in deionized water, filtered, and lyophilized to give 22 mg of yellow solid. Finally, the desired product **G4-Nal-F** (12 mg, 72%) was obtained by using semi-preparative HPLC (RP-HPLC, C18 monochromatic column, 220 nm, 25% CH_3_CN in H_2_O for 20 min with flow rate 2.0 mL/min) as yellow solid. Purity 99+% of **G4-Nal-F** was measured by using HPLC (Zorbax SB-C8, 0.8 mL/min, 254 nm, 30% CH_3_CN in H_2_O during 10 min, *t_R_* = 2.68 min). ^1^H NMR (δ, D_2_O): 1.00–1.69 (m, 36H), 1.88–1.98 (m, 3H), 2.15–2.43 (m, 10H), 2.60 (brs, 2H), 2.91–3.45 (m, 14H), 3.98–4.36 (m, 5H), 4.97–5.35 (m, 4H), 6.46–7.80 (m, 16H); MALDI-TOF-MS [M + H] ^+^ calculated for C_82_H_112_N_16_O_19_S *m/z* 1656.80, found 1657.04.

#### 2.2.13. 1-O-[6-(fluoresceinyl-5-thioureido)-hexanoyl]-2,3,4,5-(6-guanidinohexanoyl)-6-O-D-sorbitol.4HCl (G4-F)

Compound **8** (35 mg, 0.02 mmol), and FITC (8 mg, 0.02 mmol) were dissolved in 2 mL of dry THF/EtOH mixture (2:4). Et_3_N (8 μL, 0.06 mmol) was added and the solution was stirred at rt in dark under an inert atmosphere. After 16 h, the reaction mixture was concentrated, and the crude product was dissolved in 10 mL CH_2_Cl_2_. The organic layer was washed with water, dried over Na_2_SO_4_, and filtered. The resulting solid (40 mg) was dissolved in 2 mL of 1 M HCl(g) in EtOAc solution at rt and stirred for 24 h. A precipitate thus obtained was filtered, and washed with EtOAc, and CH_2_Cl_2_ several times. The solid was dissolved in deionized water, filtered, and lyophilized to give 18 mg of yellow solid. Finally, the desired compound **G4-F** (5 mg, 20%) was obtained using semi-preparative HPLC (RP-HPLC, C18 monochromatic column, 220 nm, 25% CH_3_CN in H_2_O for 20 min with flow rate 2.0 mL/min) as yellow solid. Purity 98% of **G4-F** was measured using HPLC (Kromasil-C8, 1.0 mL/min, 220 nm, 40% CH_3_CN in H_2_O during 10 min, t_R_ = 1.62 min). ^1^H NMR (δ, D_2_O): 1.15–1.57 (m, 30H), 2.19–2.36 (m, 10H), 3.01–3.12 (m, 8H), 3.54–3.65 (m, 2H), 3.98–4.47 (m, 5H), 5.05–5.51 (m, 3H), 6.54–7.23 (m, 7H), 7.49–7.65 (m, 1H), 7.90 (brs, 1H); MALDI-TOF-MS [M-H_2_O] calculated for C_61_H_86_N_14_O_15_S *m/z* 1286.61, found 1286.47.

#### 2.2.14. 1-O-6-[2-Acetamido-3-(naphthalen-2-yl)propanaminohexanoyl]-2,3,4,5-(6-guanidinohexanoyl)-6-O-D-sorbitol.4HCl (G4-Nal)

Compound **9** (40 mg, 0.02 mmol) was dissolved in 1 M HCl(g) in EtOAc solution (2 mL) at rt and stirred for 24 h. A precipitate thus obtained was filtered, and washed with EtOAc and CH_2_Cl_2_ several times. The resulting solid was dissolved in deionized water, filtered, and lyophilized to afford 23 mg of white solid. Finally, the desired compound **G4-Nal** (15 mg, 65%) was obtained after using semi-preparative HPLC (RP-HPLC, C18 monochromatic column, 220 nm, 25% CH_3_CN in H_2_O for 20 min with flow rate 2.0 mL/min) as a white solid. Purity of **G4-Nal** was measured by using HPLC (Kromasil-C8, 1.0 mL/min, 220 nm, 40% CH_3_CN in H_2_O during 10 min, *t_R_* = 2.13 min) to be +98%. ^1^H NMR (δ, D_2_O): 0.55–1.51 (m, 30H), 1.80–1.99 (m, 5H), 2.23–2.44 (m, 8H), 2.68–3.18 (m, 12H), 3.66–4.57 (m, 6H), 4.85–5.41 (m, 3H), 7.36–7.83 (m, 7H); MALDI-TOF-MS [M + H]^+^ calculated for C_55_H_90_N_14_O_13_ *m/z* 1154.68, found 1154.25.

#### 2.2.15. 4-Oxobutonoyl-[(5-aminopentyl)amino]-demethoxy geldanamycin (GA-Acid)

17-AHA-geldanamycin (5 mg, 0.01 mmol) and succinic anhydride (2.0 mg, 0.02 mmol) were dissolved in 2 mL of dry CH_2_Cl_2_. Pyridine (8 μL, 0.1 mmol) was added and the solution was stirred at rt for 18 h. 10 mL of CH_2_Cl_2_ was added to the reaction mixture and the organic layer was washed with 1 N HCl(aq), dried over Na_2_SO_4_, and filtered. The filtrate was concentrated, dried in vacuum, and the crude product was purified by semi-preparative HPLC (RP-HPLC, Eclipse XDB-C18 column, 220 nm, 10% CH_3_CN in H_2_O to 100% CH_3_CN for 20 min with flow rate 10.0 mL/min) to afford compound **GA-Acid** (5 mg, 67%) as violet color solid. ^1^H NMR (δ, MeOD): 0.98 (t, *J* = 6.5 Hz, 6H), 1.28–1.67 (m, 10H), 1.73 (s, 3H), 1.82 (brs, 1H), 2.00 (s, 3H), 2.30–2.34 (m, 1H), 2.45 (t, *J* = 7.0 Hz, 2H), 2.58 (t, *J* = 7.0 Hz, 2H), 2.72–2.76 (m, 2H), 3.17 (t, *J* = 7.0 Hz, 2H), 3.30 (s, 3H, merged with MeOD peak), 3.34 (s, 3H), 3.52–3.61 (m, 4H), 4.53 (d, *J* = 8.5 Hz, 1H), 5.21 (s, 1H), 5.59 (d, *J* = 10.0 Hz, 1H), 5.87 (t, *J* = 10.0 Hz, 1H), 6.62 (t, *J* = 12.0 Hz, 1H), 7.06 (s, 1H), 7.13 (d, *J* = 12.0 Hz, 1H); ^13 ^C NMR (δ, MeOD): 185.94, 181.13, 176.34, 174.57, 174.32, 170.89, 159.25, 146.88, 143.09, 138.10, 135.53, 134.56, 132.75, 129.75, 127.26, 109.78, 109.27, 83.21, 82.12, 74.50, 57.67, 56.97, 46.53, 40.44, 35.95, 34.70, 33.82, 31.78, 31.62, 31.06, 30.87, 30.53, 30.41, 27.69, 27.60, 22.82, 14.55, 13.71, 12.52.

### 2.3. Cell culture

HCT116, HeLa, SW480, SW620, SKOV3, CAOV3, and RAW 264.7 cells were obtained from the Korean Cell Line Bank (Seoul, Korea). Cells were maintained and cultured in serum-containing DMEM at 37 °C in a humidified incubator in the presence of 5% CO_2_ and 10% (v/v) FBS with penicillin.

### 2.4. Cellular localization studies by confocal laser scanning microscopy

HCT116 cells (1 × 10^5^ cells/well) were seeded in a 35‐mm cover glass-bottomed dish (SPL Ltd., Pocheon, Korea) and were cultured for 24 h. The media was removed, and the cells were washed with cold PBS two times. The cells were pretreated with **G4-F** (10 μM), **G4-Nal-F** (10 μM), and **G8-F** (1 μM), dissolved in 2 mL of serum-free DMEM for 30 min and were then further treated with MitoTracker (100 nM). Confocal laser scanning microscopy (Carl Zeiss LSM 710) equipped with an oil immersion lens (NA 1.30, 40×) was used to observe and analyze the intensities of fluorescence within cells. Fluorescence of the FITC was excited at 488 nm with an argon laser, and the fluorescence was observed at 550–570 nm with an emission band filter. MitoTracker, rhodamine B-dextran, and lysotracker were excited at 543 nm by a HeNe laser, and the fluorescence was observed at 600–650 nm. Pearson’s correlation coefficients and scatterplots were obtained using ImageJ software (https://imagej.nih.gov/ij) installed with a plugin, JACoP (Just Another Co-localization Plugin) (Bolte & Cordelières, 2006; Dunn et al., 2011).

HeLa cells (1 × 10^5^ cells/well) were seeded in a 35‐mm cover glass-bottomed dish, and cultured for 24 h. Live HeLa cells were pretreated with **G4-Nal-F** (10 μM) dissolved in 2 mL of serum-free DMEM for 30 min at 37 °C under 5% CO_2_/air and were stained with MitoTracker (100 nM), which is a mitochondria marker; lysotracker (150 nM), which is a lysosome marker; and rhodamine B-dextran (8.11 mM), which is an early and late endosome marker for another 30 min. To remove the non‐internalized compounds, live HeLa cells were washed with 2 mL of serum-free DMEM three times and kept in 2 mL of serum-free DMEM to keep the HeLa cells alive during observation by confocal laser scanning microscopy. The relative intensities of each fluorescence were analyzed using Zen software (Carl Zeiss, Germany).

### 2.5. Partition coefficient measurement

Partition coefficient (Log *P*) values were determined experimentally by octanol/water partitioning using the shake-flask method at neutral pH. UV–Vis spectroscopy was used to measure the concentration of the compounds according to the Beer-Lambert law. The correlation coefficient (R^2^) value was measured using a calibration curve which was plotted as a graph using the concentration of the compound in an aqueous solution and its intensity of absorption. Molecular transporters, such as **G4-Nal-F** (66.66 μg), **G4-F** (83.33 μg), and **G8-F** (500 μg) were dissolved in water (1 mL) and treated with an equal volume of octanol. The flask was shaken to distribute the compound between two phases and kept aside for 2 h to equilibrate the two layers. Aliquots from the aqueous solution were withdrawn and diluted 4-fold. The distributed concentration was measured using a UV–Vis spectrometer and Log *P* value was calculated by the formula Log *P* = Log (*P_o_*/*P_i_*), where *P_o_* is the concentration of the compound in the octanol layer, and *P_i_* is the concentration of the compound in the aqueous layer.

### 2.6. Endocytosis inhibition

HCT116 cells were pretreated with inhibitors, such as chlorpromazine hydrochloride (30 μM) for clathrin-mediated endocytosis, methyl-β-cyclodextrin (10 mM) for caveolae endocytosis, and imipramine hydrochloride (5 μM) for macropinocytosis in serum-free DMEM for 30 min at 37 °C. The cells were retreated with **G4-Nal-F** (10 μM), **G8-F** (2 μM), and maintained for another 30 min. The live cells were then analyzed using confocal laser scanning microscopy. The intensity of the fluorescence inside the cells was quantified using ImageJ software. Each analysis was performed in triplicate for accuracy.

### 2.7. Cell viability test by MTT assay

SKOV3, CAOV3, and RAW 264.7 cells were seeded in a 96‐well plate at a density of 4.5 × 10^3^ cells/well and were cultured for 24 h. The media was changed to non‐serum RPMI 1640 within 24 h. The sample solutions of **G4-Nal**, **IC**, and **GA** in non‐serum RPMI 1640 (containing 0.5% of DMSO) with different concentrations in the range of 5–25 µM were added to the cells. After incubation for 48 h, the cells were washed with cold PBS twice and exposed to MTT (3-(4,5-dimethylthiazol-2-yl)-2,5-diphenyltetrazolium bromide) for 4 h. The media was changed to DMSO, and cell viability was measured using a cell proliferation assay system. Dissolved formazan dye was quantified by measuring the absorbance at 540 nm using a plate reader. The non-treated cells were used as a control. The percentage of viable cells was calculated as the percent fluorescence intensity formed in the experimental wells relative to that in the control wells. Cell viability (%) = *A_T_*/*A_U_ X* 100 where *A_T_*, and *A_U_* represent the absorbance at 540 nm when cells were treated and untreated with samples, respectively.

## 3. Results and discussion

### 3.1. Synthesis of sorbitol-based molecular transporters

Sorbitol was selected as a scaffold since it is a carbohydrate that has six hydroxyl groups without a reducing group. In contrast to ring sugars, sorbitol’s linear structure can provide flexibility for molecular transporters, and we expect this flexibility to lead to enhanced cell membrane permeability because backbone flexibility can help facilitate interactions between the guanidiniums and anionic molecules present on the cell surface. The four secondary alcohol groups of the sorbitol were reacted to have guanidines at the terminus *via* aminocaproic acid as a linker. Thus, the molecular transporter could have a total of four guanidines, which is sufficient for intracellular permeation (Qian et al., 2013). In addition, the one primary alcohol group of the sorbitol was designed to contain naphthalene to impart lipophilicity to the molecular transporter, and the other primary group acted as a fluorescent probe for confocal microscopy analysis.

Trityl sorbitol (**2**) was prepared from α-D-glucose according to a past publication (Maiti et al., 2007). In brief, α-D-glucose was tritylated and further reduced with NaBH_4_ in MeOH to afford compound **2**. The rest of the hydroxyl groups of the sorbitol were protected using TMSCl in pyridine, and compound **3** was obtained. Unlike the previously reported methods, TMSCl was used instead of a bulkier protecting group, *tert*-butyl(chloro)diphenylsilane (TBDPSCl), since we found that TBDPSCl migrated to other hydroxyl groups during deprotection. The single TMS on the primary alcohol was removed selectively from **3** with NH_4_OAc in a MeOH/CH_2_Cl_2_ mixture (6:1) to afford **4,** which was acylated sequentially with *N*-Cbz-protected aminocaproic acid. After removing the other TMS protecting groups with TBAF in dry THF, the sorbitol was peracylated with *N′*,*N″*-bis-*tert*-butoxycarbonyl-guanidino hexanoicacid (**Compound A**) in the presence of DMAP, and EDC in dry CH_2_Cl_2_ to afford **7**. Hydrogenolysis of **7** removed the Cbz-protecting group to produce **8**, and then **Nal** (Scheme S1) was conjugated to the amino group of **8**. For conjugating naphthalene to the molecular transporter, naphthalene was substituted with acetylalanine, which facilitates conjugation to the amine of the molecular transporter. Thus, naphthalene was conjugated successfully to afford **9**. The trityl group of **9** was removed, and *N*-Cbz-protected aminocaproic acid was acylated again to give **11**. After deprotection of **11** by hydrogenolysis, **12** was treated with fluorescein-5-isothiocyanate (FITC) and Et_3_N in THF/EtOH to afford a fluorescence-labeled molecular transporter. The compound was treated with 1 M HCl(g) in EtOAc for 24 h to remove all Boc groups at guanidines. The resulting molecular transporter, which has four guanidines and a fluorescence tag (**G4-Nal-F**), was purified to +99%.

**Scheme 1.**
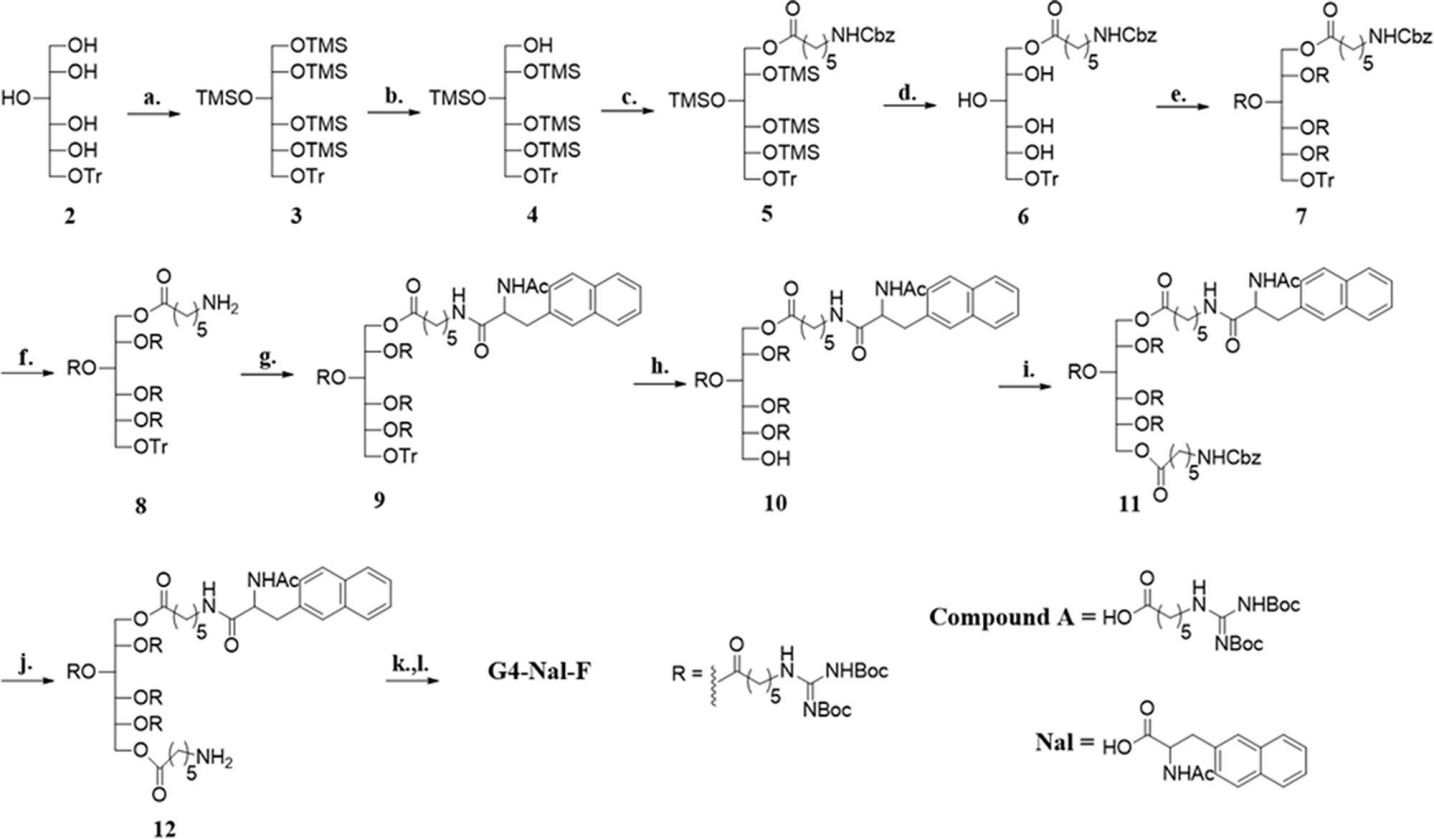
Synthesis of **G4-Nal-F**: (a) TMSCl, pyridine, 0 °C → rt, 2 days, (b) NH_4_OAc, MeOH/CH_2_Cl_2_ (6:1), rt, 12 h, (c) *N*-Cbz aminohexanoic acid, EDC, DMAP, dry CH_2_Cl_2_, rt, 12 h, (d) TBAF, dry THF, ice bath, 1 h, (e) **Compound A**, EDC, DMAP, molecular sieves powder 4 Å, dry CH_2_Cl_2_, rt, 3 days, (f) H_2_(g), 30 psi, 10% Pd/C, MeOH/CH_2_Cl_2_ (5:2), 6 h, (g) **Nal**, EDC, DMAP, dry CH_2_Cl_2_, rt, 1 day, (h) 2.5% TFA in CH_2_Cl_2_, −15 → 0 °C, 15 min, CH_2_Cl_2_, (i) *N*-Cbz aminohexanoic acid, EDC, DMAP, dry CH_2_Cl_2_, rt, 2 days, (j) H_2_(g), 30 psi, 10% Pd/C, 24 h, MeOH/CH_2_Cl_2_ (5:2), (k) FITC, Et_3_N, THF/EtOH (2:4), 16 h, rt, (l) 1 M HCl(g) in EtOAc, rt, 24 h.

To evaluate the mitochondria-targeting ability and lipophilic effects, we synthesized other versions of molecular transporters. Compound **G4-F** was prepared with a similar structure to **G4-Nal-F** but without naphthalene (Figure 1(b), Scheme S2). To deliver a drug to mitochondria using a molecular transporter, **G4-Nal** without a fluorescence tag was also prepared using similar methods (Figure 1(c), Scheme S3). Finally, to examine the effect of the number of guanidines on mitochondria targeting, a molecular transporter with eight guanidines and a fluorescence tag (**G8-F**) was also synthesized according to previous literature (Figure 1(d)) (Maiti et al., 2007).

All final products were purified by prep RP-HPLC using a C18 monochromatic column. All compounds were characterized using various analytical techniques. The purity of products was assessed by RP-HPLC equipped with an analytical C8 or C18 monochromatic column. The authenticity of the final compounds was confirmed by MALDI-TOF mass spectrometry (Figures S1–S4).

### 3.2. Cellular uptake and intracellular localization of sorbitol-based molecular transporters

**Figure 2.**
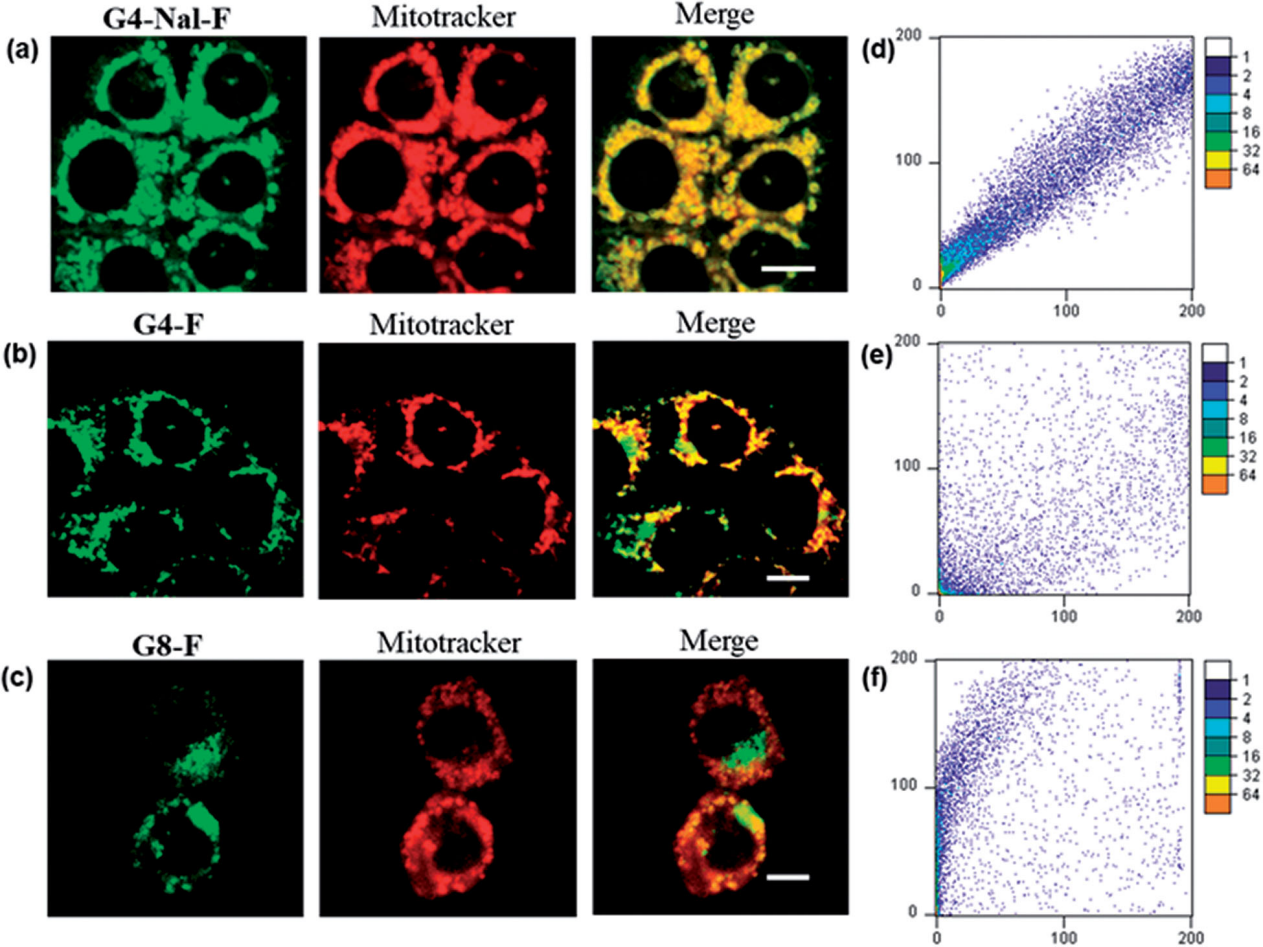
Mitochondrial colocalization studies using confocal laser scanning microscopy in HCT116 cells. (a) **G4-NAL-F** (10 μM), (b) **G4-F** (10 μM), and (c) **G8-F** (1 μM) were incubated for 30 min and then with MitoTracker (100 nM) for 30 min. Green and red fluorescence indicate localization of molecular transporter and MitoTracker, respectively. (Scale bar: 10 μm) (d–f) Scatterplots of intensities of green and red pixels from the same spots in each fluorescent image. The green intensities are used as the x-coordinate and the red intensities as the y-coordinate. The frequencies of pixels appearing in the scatterplot were distinguished by colors, and the numbers beside the bar indicate the frequency level.

The synthesized molecular transporters were tested for cellular uptake and intracellular localization. An HCT116 cell line derived from human colon cancer was cultured and treated, respectively with **G4-Nal-F** (10 µM), **G4-F** (10 µM), and **G8-F** (1 µM) dissolved in serum-free media. The **G8-F** concentration was decreased to 1 µM since we found that some cells were dead due to cytotoxicity in 10 µM. Conversely, **G4-Nal-F** and **G4-F**, which have four guanidines, did not have negative effects on cell viability, confirming that the number of guanidines can affect the inherent toxicity of the molecular transporter. Previously, it was established that fewer guanidine groups within the same scaffold led to less cytotoxicity than a compound with more guanidine groups (Im et al., 2013). After incubation with molecular transporters for 30 min, the cells were co-incubated for a further 30 min with MitoTracker (100 nM) for mitochondria staining. Then, the cells were washed with fresh cell media three times to remove any uninternalized compounds. The live cells were examined visually through confocal laser scanning microscopy (Carl Zeiss LSM 710), exhibiting high green fluorescence inside cells (Figures 2(a–c)). This indicated that all molecular transporters were well-internalized within 30 min, and four guanidine groups were sufficient for cellular uptake. To evaluate how strongly each molecular transporter targeted mitochondria, green fluorescence images and MitoTracker images were obtained independently and then combined. According to the merged images, **G4-Nal-F** showed the best localization to mitochondria.

Additionally, colocalization was evaluated using scatterplots where the green fluorescence intensity of molecular transporters was plotted against the red fluorescence intensity of the MitoTracker for all individual pixels in each merged image (Bolte & Cordelières, 2006; Dunn et al., 2011). In each scatterplot, the x-axis is the intensity of the green fluorescence signal, while the y-axis is the MitoTracker. As shown in Figure 2(d), there is a proportional co-distribution from the two signals, showing how the dots of the cluster exist around a straight line. This means that the signals appear with similar intensities at every point inside the cells. In contrast, in Figures 2(e,f), the distributions of points are random, with two signals appearing with different intensities at the same point inside cells. This demonstrates that **G4-Nal-F** has a high preference for mitochondrial localization, whereas neither **G4-F** nor **G8-F** performs as well as **G4-Nal-F**.

Furthermore, the co-localization degree of each merged image was evaluated by calculating Pearson’s correlation coefficient (PCC), which can quantify the degree of overlap between two parameters (Table 1). The PCC ranges from −1 to +1, and if the two fluorescence channels correlate perfectly, i.e. complete colocalization, the PCC value would be +1. The PCC of **G4-Nal-F** was 0.976, which reflects the highest colocalization with MitoTracker among the molecular transporters. However, the PCC values of **G4-F** and **G8-F** were 0.877 and 0.693, respectively, indicating that both have a weak preference for mitochondria.

**Table 1.** Partition coefficient (Log *P*) and Pearson’s correlation coefficient values of the molecular transporters.

Compound name	Partition coefficient (Log *P*)	Pearson’s correlation coefficient
**G4-Nal-F**	−0.880	0.976
**G4-F**	−1.876	0.877
**G8-F**	−1.596	0.693

To address the effect of lipophilicity for mitochondria targeting, the Log *P* of each molecular transporter was measured by the shake-flask method using water and octanol for partitioning. The experiment was performed at neutral pH where all the guanidines of the molecular transporters were present as guanidiniums (Figure S5). Thus, all the cationic molecular transporters existed predominantly in the water layer, resulting in negative Log *P* values (Table 1, Table S1). As expected, the Log *P* of **G4-Nal-F** was −0.880 and the highest among the molecular transporters, since **G4-Nal-F** contained alanine-naphthalene. The lack of a lipophilic group led to more negative Log *P* values for **G4-F** and **G8-F** (−1.876 and −1.596, respectively). According to the Log *P* values, a single lipophilic group, i.e. alanine-naphthalene, can provide **G4-Nal-F** with ∼10 times more hydrophobicity than other molecular transporters.

Therefore, the partition coefficients and PCC values confirmed that **G4-Nal-F** is hydrophobic among the molecular transporters and shows the highest localization in mitochondria. It is evident that a lipophilic group is essential for efficient mitochondria targeting. Thus, **G4-Nal-F** was chosen for the following experiments.

### 3.3. Subcellular localization and cellular uptake mechanism

**Figure 3.**
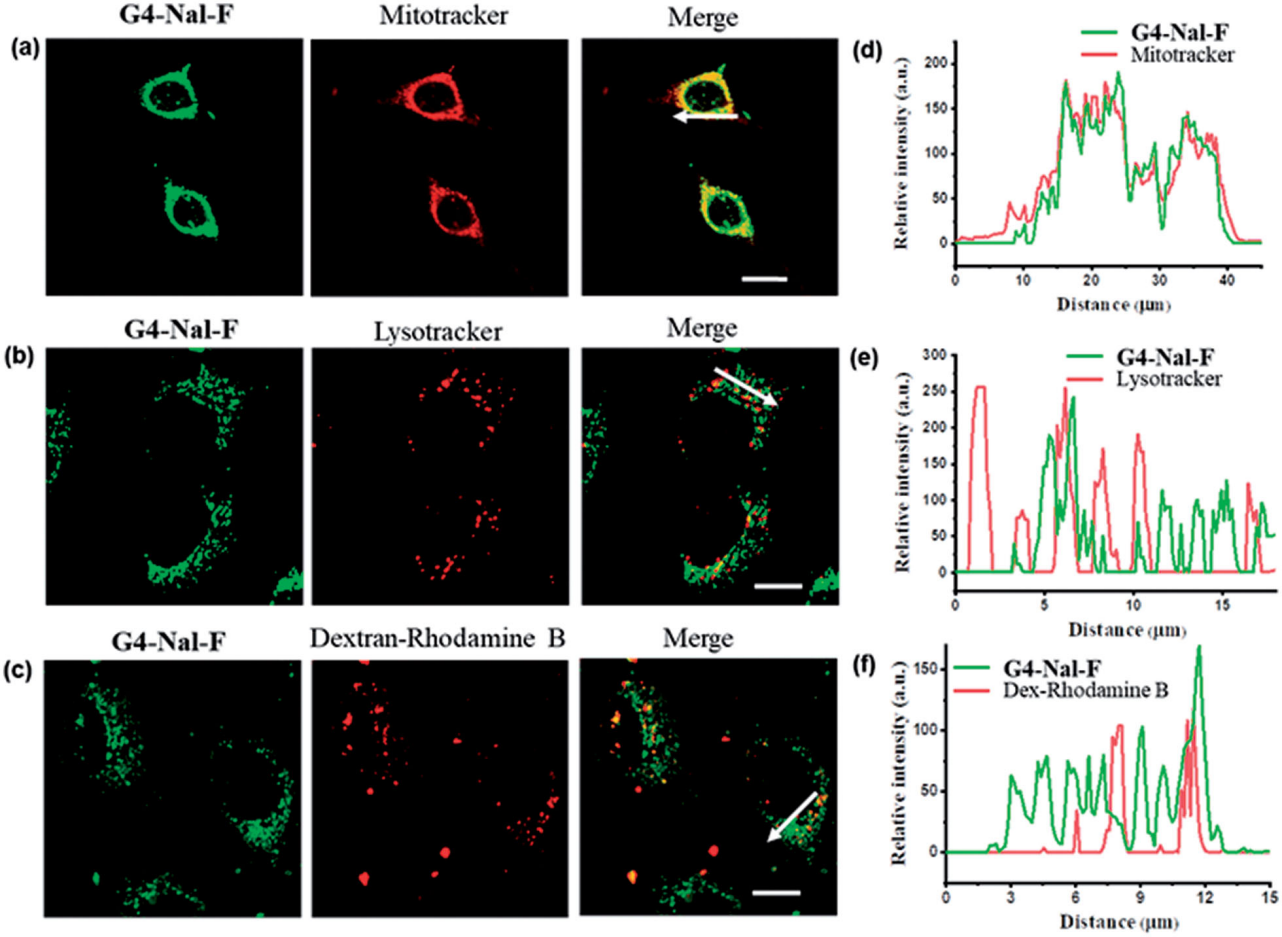
Subcellular localization study in live HeLa cells by incubating **G4-Nal-F** (10 µM) with subcellular markers: (a) MitoTracker (100 nM), (b) lysotracker (100 nM), (c) dextran-rhodamine B (1 mg/mL), (scale bar: 10 μm). (d–f) The relative intensities of each fluorescence signal along the arrow are shown.

To examine the subcellular localization of the molecular transporter in detail, we performed experiments with another cell line, HeLa. Live HeLa cells were incubated with 10 µM of **G4-Nal-F** for 30 min and then for another 30 min with one of several specific organelle markers: MitoTracker (100 nM), lysotracker (150 nM) for lysosome staining, and rhodamine B-dextran (1 mg/mL) for endosome staining. After rinsing the cells with cell media, **G4-Nal-F** showed significant colocalization with MitoTracker on confocal microscopy images (Figure 3(a)). In contrast, coincubation of **G4-Nal-F** with either lysotracker or dextran-rhodamine B showed little or no colocalization in images (Figures 3(b,c)). The intensity profiles of fluorescent images were also measured within the cells. The relative intensity of each signal was recorded according to a line through cells (Figures 3(d–f)), supporting that **G4-Nal-F** has high selectivity toward mitochondria. In other studies, it was reported that compounds containing multiple guanidines showed poor localization in mitochondria or localized in several organelles in addition to mitochondria, such as lysosomes and endoplasmic reticulum (Sibrian-Vazquez et al., 2008; Ghosh et al., 2010; Hamill et al., 2016). Therefore, it is clear that guanidines are necessary for efficient cellular uptake, but this does not assure selective localization in mitochondria. In this study, the molecular transporter designed to have guanidines and a lipophilic group achieved both plasma membrane permeability and mitochondria targeting.

**Figure 4.**
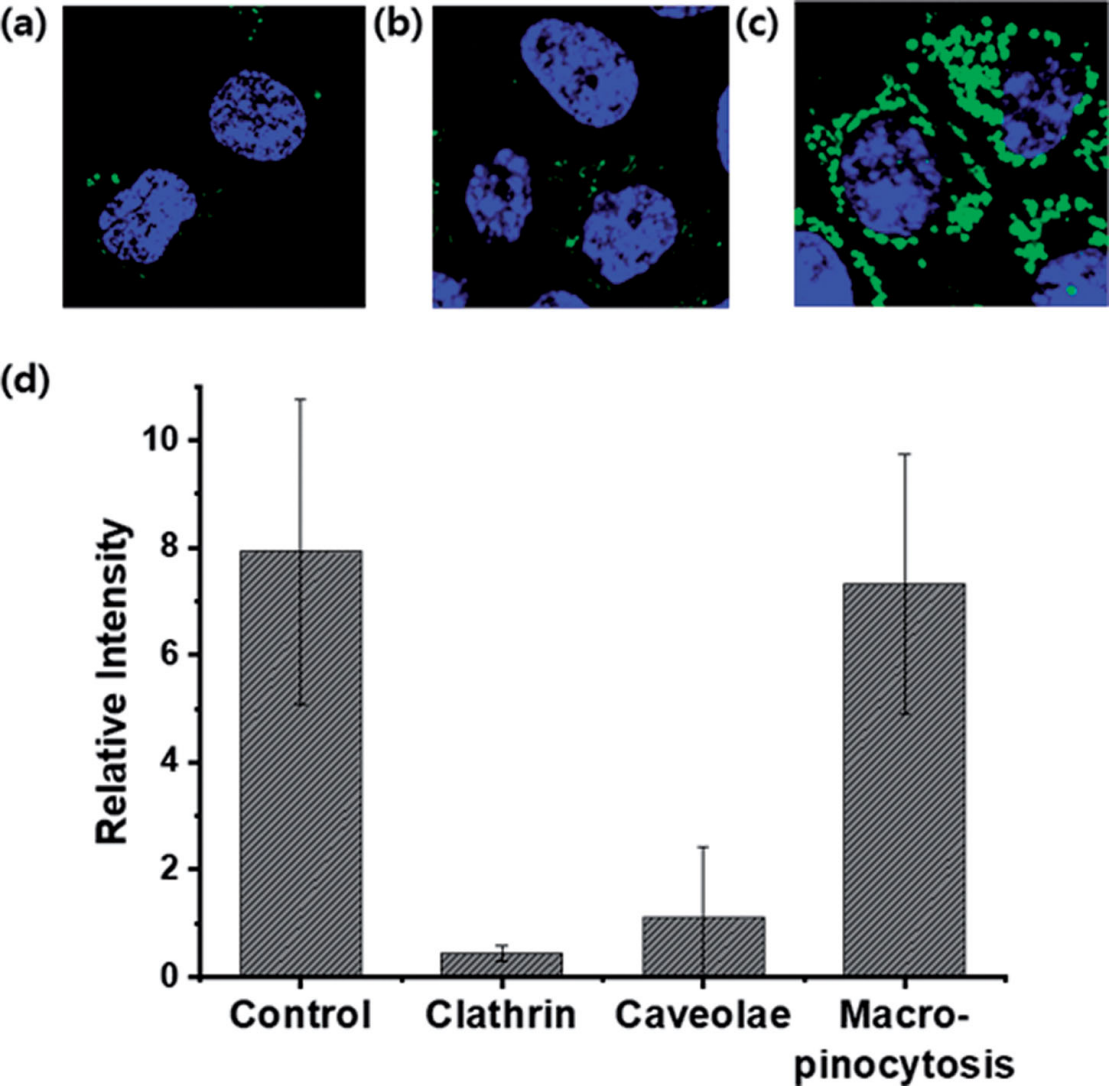
Study of the cellular uptake mechanism of **G4-Nal-F** (10 µM) in HCT116 cells using endocytosis inhibitors. (a) Clathrin-mediated endocytosis inhibition by chlorpromazine (30 μM), (b) caveolae-mediated endocytosis inhibition by methyl-β-cyclodextrin (10 mM), (c) macropinocytosis inhibition by imipramine hydrochloride (5 μM), (d) comparison of relative fluorescence intensity of **G4-Nal-F** under different inhibition conditions. **G4-Nal-F** incubation without an inhibitor is set as the control. The nucleus (blue color) was stained with Hoechst 33342 (Scale bar: 20 μm).

In previous studies, the cellular uptake of **G8-F** was proposed to involve energy-dependent endocytosis. It was also reported that **G8-F** could not internalize into HeLa cells at low temperatures (4 °C) or under ATP-depleted conditions (Im et al., 2011). To obtain more insight into the cellular uptake mechanism of the molecular transporter, **G4-Nal-F** was co-incubated in HCT116 cells with diverse endocytosis inhibitors. The cells were treated with imipramine hydrochloride (5 μM) for inhibition of macropinocytosis, chlorpromazine hydrochloride (30 μM) for clathrin-mediated endocytosis, or methyl-β-cyclodextrin (10 mM) for caveolae-mediated endocytosis. Chlorpromazine is a cationic amphipathic drug that can incorporate easily into lipid bilayers and block invaginations from the large membrane to inhibit the formation of clathrin-coated pits (Im et al., 2011). Methyl-β-cyclodextrin has a high affinity toward plasma membrane cholesterol and intensively depletes cholesterol from plasma membranes to inhibit caveolae-mediated endocytosis (Im et al., 2017). Imipramine hydrochloride inhibits the formation of membrane ruffling, which is a crucial step to initiate macropinocytosis (Lin et al., 2018; Singh et al., 2021). After 30-min incubation with inhibitors, the cells were retreated with **G4-Nal-F** (10 µM) for an additional 30 min. The cells were washed and observed by confocal laser microscopy, showing that the cellular uptake degree was dependent on the inhibitors. Cells treated without inhibitors were used as a control. The cellular uptake of **G4-Nal-F** was greatly reduced when chlorpromazine hydrochloride and methyl-β-cyclodextrin were used, showing that both clathrin-mediated endocytosis and caveolae-mediated endocytosis are involved in cellular uptake (Figures 4(a,b)). However, there was no inhibition of cellular uptake when imipramine hydrochloride was used, which suggests that macropinocytosis is not a major mechanism for internalization of **G4-Nal-F** (Figure 4(c)). Next, the cellular uptake mechanism of **G8-F** was also investigated. The cells were treated with each inhibitor in the same way followed by **G8-F** (2 μM) treatment. The green fluorescence inside cells was greatly reduced when the cells were treated with chlorpromazine, indicating that clathrin-mediated endocytosis was involved in cellular uptake (Figure S6). There was partial inhibition of cellular uptake by imipramine, indicating that macropinocytosis was also involved to some extent. The green fluorescence intensities of cells were quantified using ImageJ software, and their relative intensities based on inhibition type are displayed in Figure 4(d) and Figure S6(d) for comparison. These results contrast with previous reports on the cellular uptake of **G8-F/GFP** complex, which suggested cellular uptake mostly relies on macropinocytosis (Im et al., 2017). We confirmed that, as reported in other publications on CPPs and molecular transporters, cellular uptake processes are highly dependent on the structures, molecular weights, charges, and cargoes of delivery vectors (Maiolo et al., 2005; Stanzl et al., 2013).

### 3.4. Delivering drugs to mitochondria via the molecular transporter

Next, we tested the mitochondria drug targeting capability of the molecular transporter. Since the fluorescent probe was no longer necessary and needed to be replaced by a drug, **G4-Nal** without FITC was used. First, *in vitro* stability of **G4-Nal** in PBS and human plasma was studied. As shown in Figure S7, the percentage of intact **G4-Nal** was 93.6% in PBS and 81.3% in human plasma, demonstrating sufficient stability to reach mitochondria. Geldanamycin (**GA**) was used as a model cargo drug. **GA** is a well-known antitumor antibiotic that inhibits the function of heat shock protein 90 (Hsp90), which is a potential target for cancer therapy (Franke et al., 2013). Since it belongs to a family of molecular chaperones, Hsp90 helps stabilize numerous proteins inside cells to protect protein folding, preventing the apoptosis process. Moreover, mitochondrial Hsp90s are involved in cancer signaling networks that promote tumor development and metastasis (Kang et al., 2009). Since Hsp90s are overexpressed in cancerous cells, inhibition of Hsp90 in mitochondria can prevent disease progression and block the antiapoptotic nature of Hsp90.

**Figure 5.**
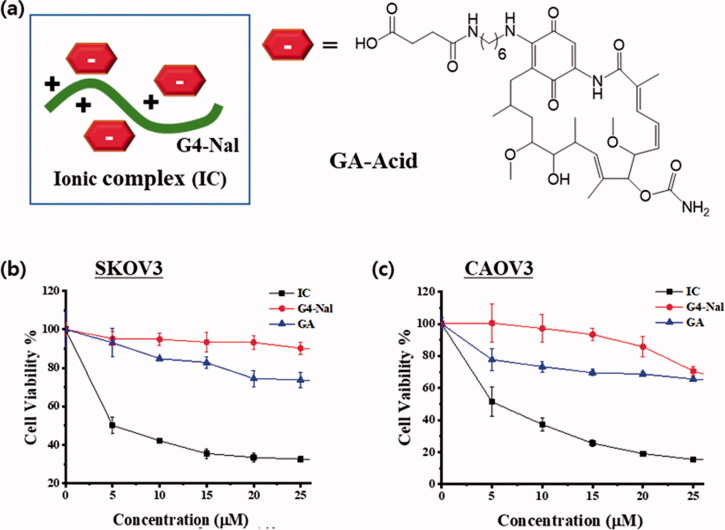
(a) Graphical representation of the **IC** made by electrostatic interactions between **G4-Nal** and **GA-Acid**. Cell viability of **G4-Nal**, **GA**, and **IC** in (b) SKOV3 and (c) CAOV3 cell lines, based on the MTT assay. **GA**: geldanamycin, **IC**: ionic complex between **G4-Nal**/**GA-Acid** in (1:3) composition.

Until recently, other strategies for targeting mitochondria, such as delocalized lipophilic cations, mitochondria penetrating peptides, and guanidine-based vehicles have relied on covalent conjugation for attaching and delivering cargo. Instead of covalently attaching a drug to the molecular transporter, a drug was bound to the vehicle through ionic bonds. To this end, a geldanamycin derivative (**GA-Acid**) with a carboxylic acid was prepared (Scheme S4). It was confirmed in a previous study that modification at C17 in the structure of **GA** does not hamper the binding affinity of **GA** toward Hsp90 (Shen et al., 2005; Franke et al., 2013). Thus, it was expected that an ionic complex is formed by electrostatic interactions between the positively-charged guanidiniums of **G4-Nal** and the negatively-charged carboxylates of **GA-Acid**s. In the case of covalent bonding with a drug, extra synthetic work is required, and a vehicle generally can carry only a single drug. The ionic complex has great advantages in that a single vehicle can carry multiple drugs at once, and the process of preparation is simple. Herein, we prepared the ionic complex (**IC** hereafter) with **G4-Nal** and **GA-Acid** in a ratio of 1:3 (Figure 5(a)). Both **GA** and **GA-Acid** have very poor aqueous solubility, limiting their clinical success. Interestingly, when **GA-Acid** was simply mixed with **G4-Nal**, the resulting **IC** along with the drugs were soluble in water. The aqueous stability of **IC** was stable, showing over 87% of the conjugate after 21 h of incubation (Figure S8). Then, the cell lines of SKOV3 and CAOV3 (human ovarian cancer cell lines) were treated with **IC**, **G4-Nal**, or **GA** at different concentrations (0–25 µM). After incubation for 48 h at 37 °C, cell viability was measured based on MTT assay. Notably, **IC** showed the greatest reduction in cell viability in both cell lines while **GA** appeared to be much less toxic (Figures 5(b,c)). In particular, the IC_50_ values of **IC** were estimated to be 6.61 µM in SKOV3, and 4.51 µM in CAOV3 cells. In contrast, the reduction of cell viability by 25 µM of **GA** was only 26% in SKOV3, and 35% in CAOV3. In addition, **G4-Nal** appeared to be non-toxic at 25 µM in SKOV3 and 20 µM in CAOV3. Cell viability testing was also conducted using the same methods on mouse macrophage RAW 264.7 cells, which are normal cells. Cell viability was reduced to 21% by **IC**, whereas **GA** showed 64% viability at 25 µM concentration of each compound (Figure S9). The IC_50_ value of **IC** in RAW 264.7 was estimated to be 9 µM, while that of **GA** was over 35 µM. Unfortunately, the cytotoxicity of **IC** was not significantly different between cancer and normal cells. These results demonstrate that **IC** was translocated well to the highly impermeable mitochondria, along with the cargo (**GA-Acid**), inhibiting Hsp90 and exhibiting cytotoxicity in cells. The drug **GA** had poor solubility in media and provided far less cytotoxicity than **IC**, whereas **G4-Nal** showed negligible cytotoxicity. Therefore, it is apparent that the anticancer effects of **IC** were derived from the drug rather than the molecular transporter, which plays the role in delivering and targeting. Another benefit is that drugs or their derivatives that have low aqueous solubility can exhibit water solubility after forming an ionic complex with a molecular transporter. We expect mitochondria targeting using this molecular transporter can be applied to anticancer therapy.

## 4. Conclusion

Mitochondria are involved in many pathologies due to their crucial roles in energy generation, cellular metabolism, and cellular apoptosis. Therapeutic investigations based on mitochondria targeting are providing exciting new approaches to disease treatment and drug discovery. Thus, various strategies for mitochondrial drug targeting are being developed. In the present study, we synthesized sorbitol-based molecular transporters that could target mitochondria. We confirmed by Log *P* measurement that alanine-naphthalene increased the hydrophobicity of the molecular transporter. In comparison with other versions of molecular transporters, the molecular transporter **G4-Nal-F** was verified to be the most efficient mitochondria-targeting compound. The addition of a lipophilic group can promote localization of the compound to the mitochondria with a high degree of specificity. In addition, a geldanamycin derivative was successfully delivered to mitochondria after the formation of an ionic complex with the molecular transporter. The use of a molecular transporter allows less drug to be used for treatment, and this can decrease the side effects of the drug. As a drug delivery vehicle, the molecular transporter can carry various types of cargo, such as therapeutic proteins, peptides, polymers, nanoparticles, and insoluble small molecules to mitochondria.

Several recent studies indicated that mitochondrial dysfunction plays a pivotal role in the pathogenesis of neurodegenerative diseases. Like other molecular transporters that are blood-brain barrier (BBB) permeable, we expect our molecular transporter to have the capability to translocate across the BBB to reach the mitochondria in the brain (Im et al., 2011; Jin et al., 2011; Im et al., 2013; Biswas et al., 2017; Lee et al., 2017). Thus, future applications of mitochondria targeting by this molecular transporter should be explored for the treatment of major neurodegenerative diseases. Furthermore, efficient mitochondria targeting will be useful for the treatment of drug-resistant cancer cells because the targeted drug can directly act on a specific system, such as apoptosis in mitochondria. Since mitochondria are attractive therapeutic targets for chemotherapy, an investigation of the usefulness of molecular transporters for delivering other pharmacological agents to mitochondria is underway.

## Supplementary Material

Supplemental Material
