# Supplementary material for: Mitochondria targeting molecular transporters: synthesis, lipophilic effect, and ionic complex
Source: Drug Deliv. 2022 Jan 11;29(1):270–83. doi: 10.1080/10717544.2021.2023696 (PMC8757599; doi:10.1080/10717544.2021.2023696)
Supplement: Supplemental Material [file IDRD_A_2023696_SM3053.docx]

**Supplementary Information**

**Mitochondria Targeting Molecular Transporters: Synthesis, Lipophilic Effect, and Ionic Complex**

Akula S. N. Murthy^a^, Sanket Das^b^, Tejinder Singh^a^, Tae-Wan Kim^c^, Nasim Sepay^a^, Seob Jeon^d^, and Jungkyun Im^a,e*^

^a^ Department of Electronic Materials and Devices Engineering, Soonchunhyang University, Asan, 31538, Republic of Korea

^b^ Department of Chemistry, Pohang University of Science and Technology, 77 Cheongamro, Pohang, 37673, Republic of Korea

^c^ Department of Medical Life Science, Soonchunhyang University, Asan, 31538, Republic of Korea

^d^ Department of Obstetrics and Gynecology, College of Medicine, Soonchunhyang University Cheonan Hospital, Cheonan, 31151, Republic of Korea

^e^ Department of Chemical Engineering, Soonchunhyang University, Asan, 31538, Republic of Korea

*****Correspondence should be addressed to Prof. Jungkyun Im: jkim5279@sch.ac.kr

**Contents**

1. **Synthesis**
2. **Spectral data of the molecular transporters**
3. **Partition coefficients of the molecular transporters**
4. **Cellular uptake mechanism study**
5. **Stability of G4-Nal, and IC**
6. **Cell viability test by MTT assay**

**1. Synthesis**


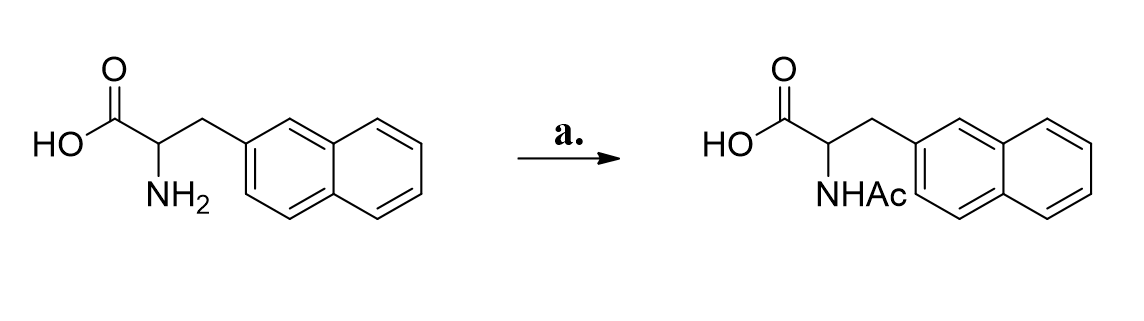


**Scheme S1**. Synthesis of **Nal**: (a) Ac_2_O, NaOH, dioxane, H_2_O, 0 °C🡪 rt, 16 hr.

**2-Acetamido-3-(naphthalen-2-yl)-propanoic acid (Nal)**

3-(2-naphthyl)-DL-alanine (30 mg, 0.14 mmol) was dissolved in 2 mL of dry dioxane and allowed to cool to 0 °C. 2 mL of 4N NaOH(aq) solution was added dropwise under cold condition and continued the stirring for additional 15 min at 0 °C. To a reaction mixture, Ac_2_O (0.04 mL, 0.41 mmol) was added drop wise at 0 °C. After addition of Ac_2_O, the reaction temperature was slowly raised to rt. Progress of the reaction was monitored by TLC. After completion of reaction, the reaction mixture was evaporated to half of its volume and adjusted the pH to neutral with 1N HCl(aq) solution. Then the mixture was diluted with CH_2_Cl_2_ and washed with 10% NaHCO_3_(aq) solution. The organic layer was dried over Na_2_SO_4_, filtered, and concentrated in vacuum to afford the product (Scheme S1**)**, which was purified by column chromatography (using 70-230 mesh size silica with CombiFlash RF+ Lumen instrument with an integrated UV detector (MA, USA); 40% MeOH in CH_2_Cl_2_ as mobile phase) to give compound **Nal** (57 mg, 96%) as foamy pale white solid. ^1^H NMR (CDCl_3_): δ 1.85 (s, 3H), 3.09-3.11 (m, 1H), 3.34-3.39 (dd, 1H), 4.59-4.62 (m, 1H), 7.36-7.44 (m, 3H), 7.67-7.72 (d, 2H), 7.74-7.76 (m, 2H).


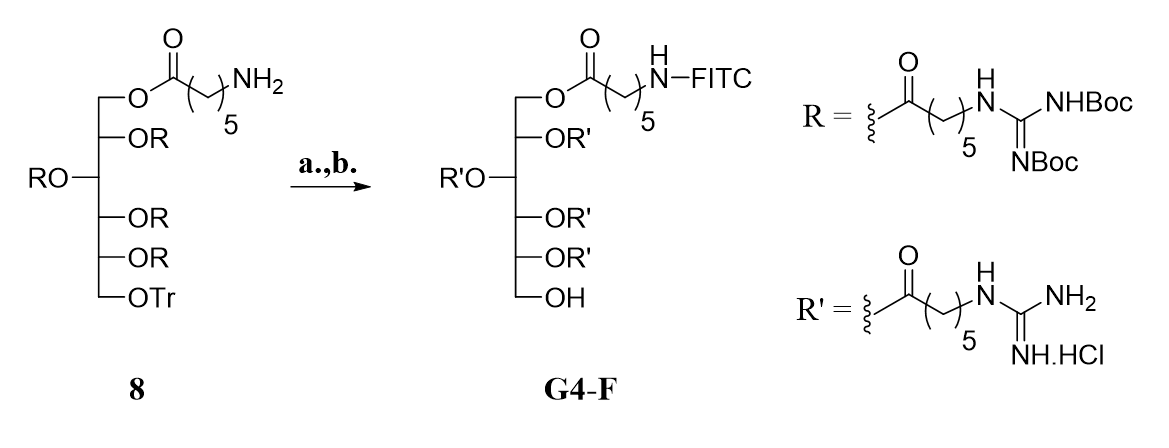


**Scheme S2.** Synthesis of **G4-F**: (a) FITC, Et_3_N, EtOH/THF (2:4), rt, 16 hr in dark, (b) 1M HCl(g) in EtOAc, rt, 24 hr in dark.


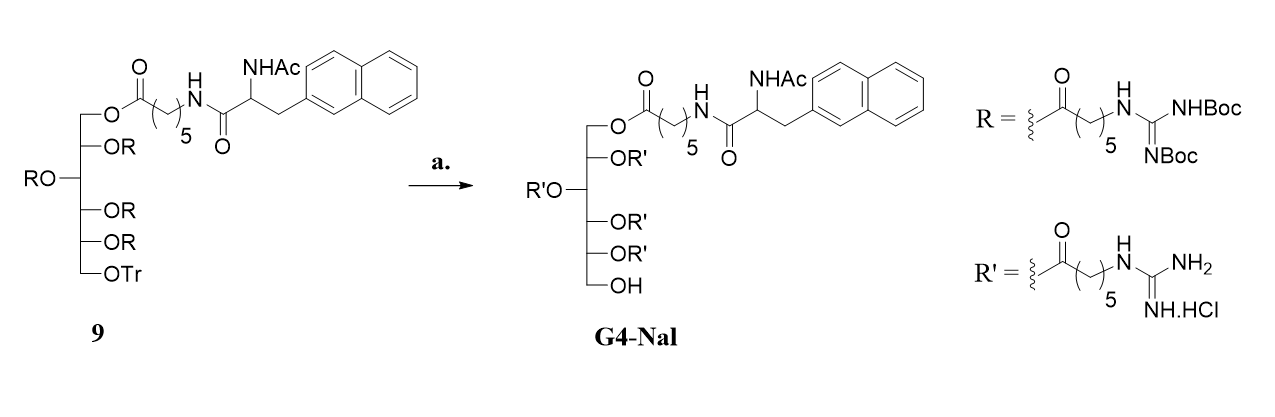


**Scheme S3**. Synthesis of **G4-Nal**: (a) 1M HCl(g) in EtOAc, rt, 24 hr.

**Scheme S4.** Synthesis of **GA-Acid**: (a) succinic anhydride, pyridine, CH_2_Cl_2_, rt, 18 hr.

**2. Spectral data of the molecular transporters**

| (a) | 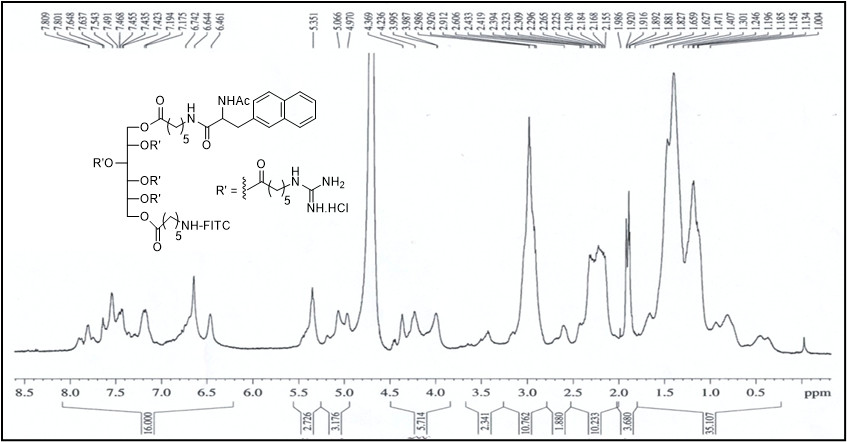 |
| --- | --- |
| (b) | 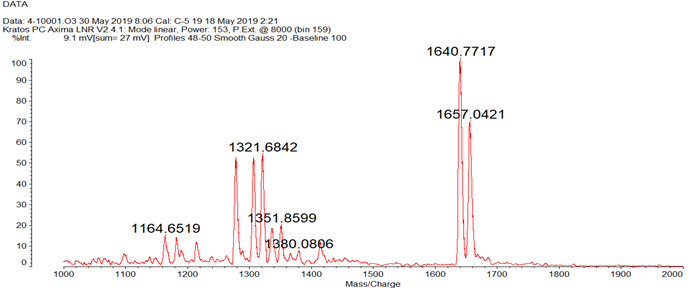 |
| (c) | 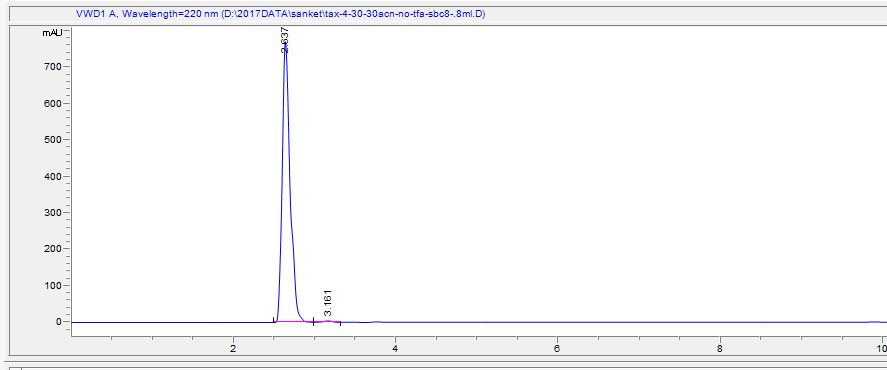 |

**Figure S1.** Characteristic data of **G4-Nal-F**. (a) ^1^H-NMR spectrum, (b) MALDI-TOF-MS [M+H] ^+^ data: *m/z* calculated for C_82_H_112_N_16_O_19_S 1656.80, found 1657.04, (c) RP-HPLC analysis: analytical HPLC (C18 monochromatic RP column, 220 nm, 2 mL/min, 25% ACN in H_2_O for 20 min, t_R_ = 2.68 min, showed the purity > 99%.

| (a) | 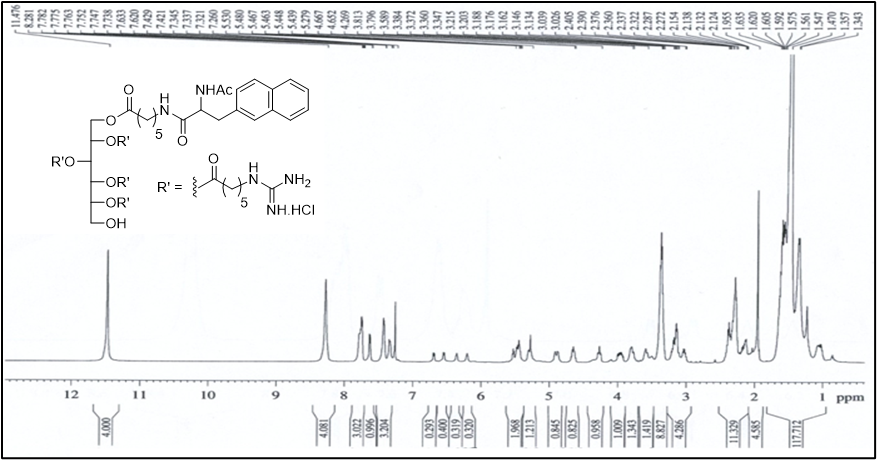 |
| --- | --- |
| (b) | 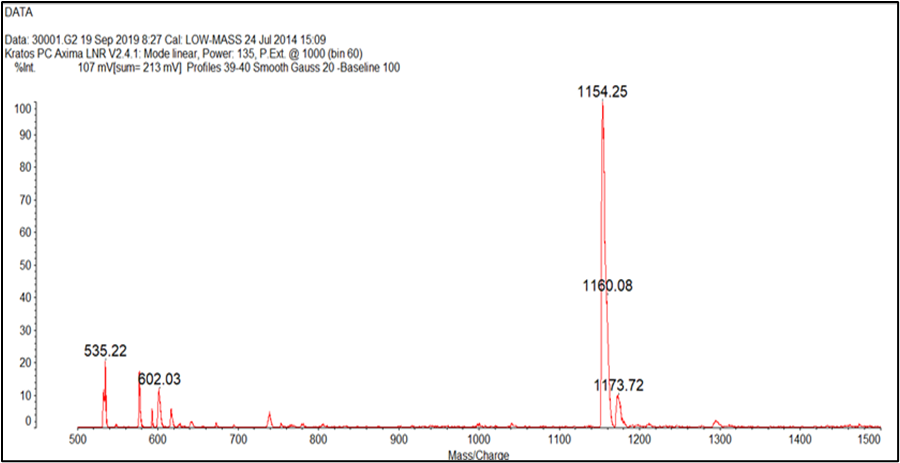 |
| (c) | 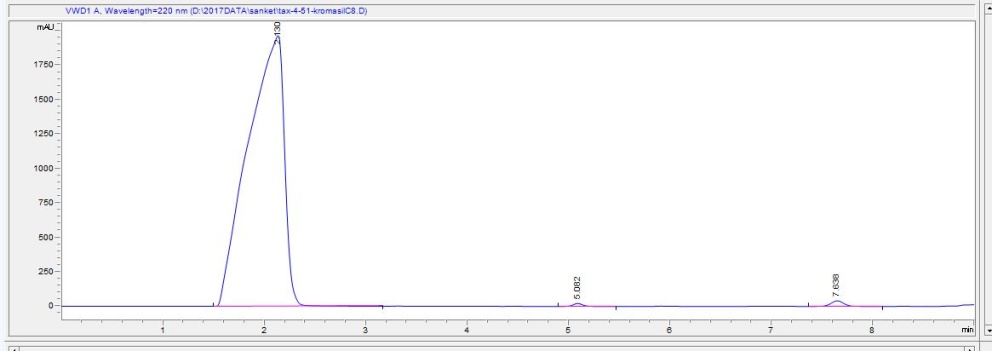 |

**Figure S2.** Characteristic data of **G4-Nal**. (a) ^1^H-NMR spectrum, (b) MALDI-TOF-MS [M+H] ^+^ data: *m/z* calculated for C_55_H_90_N_14_O_13_ 1154.68, found 1154.25, (c) RP-HPLC analysis: analytical HPLC (C18 monochromatic RP column, 220 nm, 2 mL/min, 25% ACN in H_2_O for 20 min, t_R_ = 2.13 min, showed the purity > 98%.

| (a) | 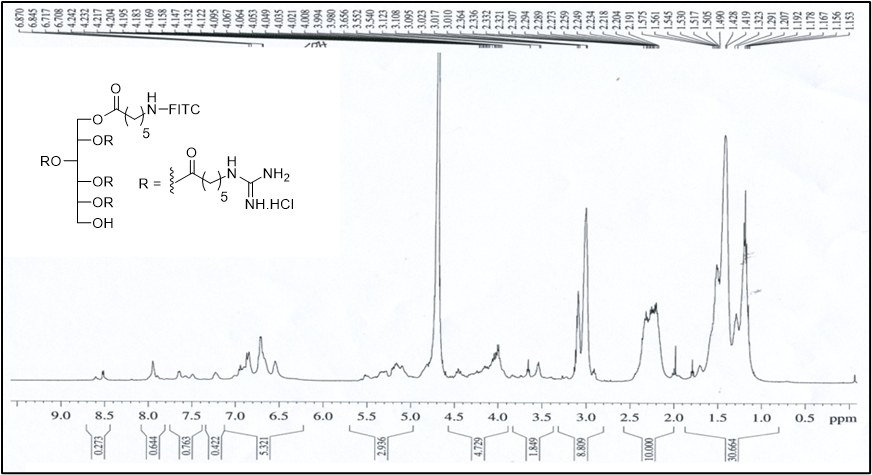 |
| --- | --- |
| (b) | 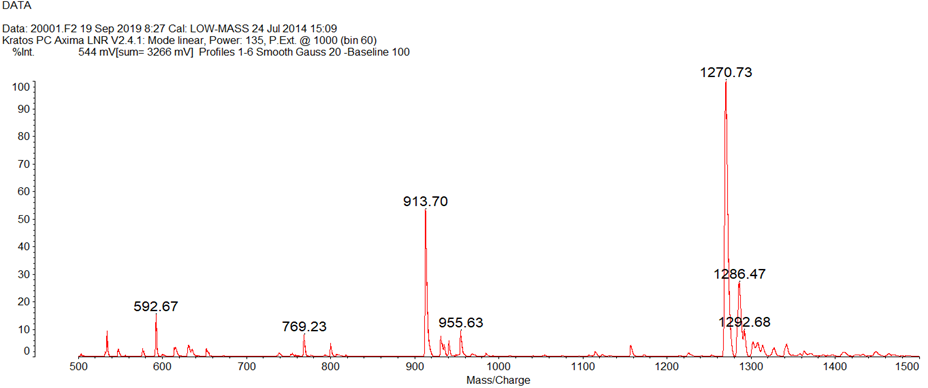 |
| (c) | 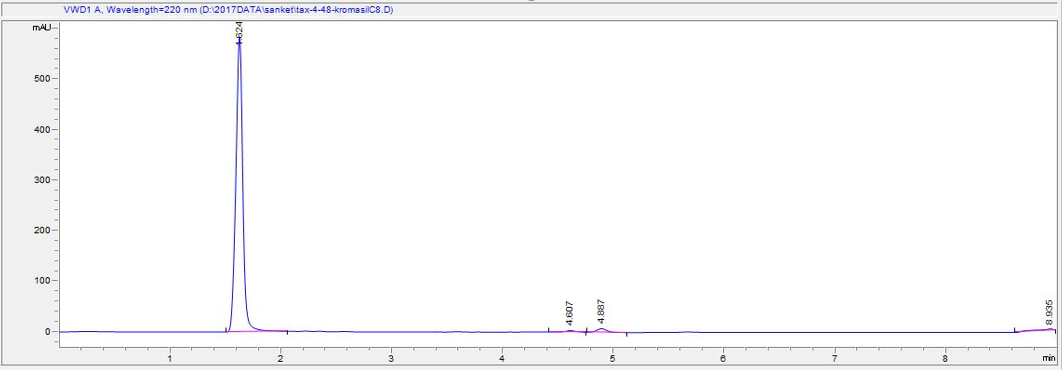 |

**Figure S3.** Characteristic data of compound **G4-F**. (a) ^1^H-NMR spectrum, (b) MALDI-TOF-MS [M-H_2_O] data: *m/z* calculated for C_61_H_86_N_14_O_15_S 1286.61, found 1286.47, (c) RP-HPLC analysis: analytical HPLC (C18 monochromatic RP column, 220 nm, 2 mL/min, 25% ACN in H_2_O for 20 min, t_R_ = 1.62 min, showed the purity > 98%.

**
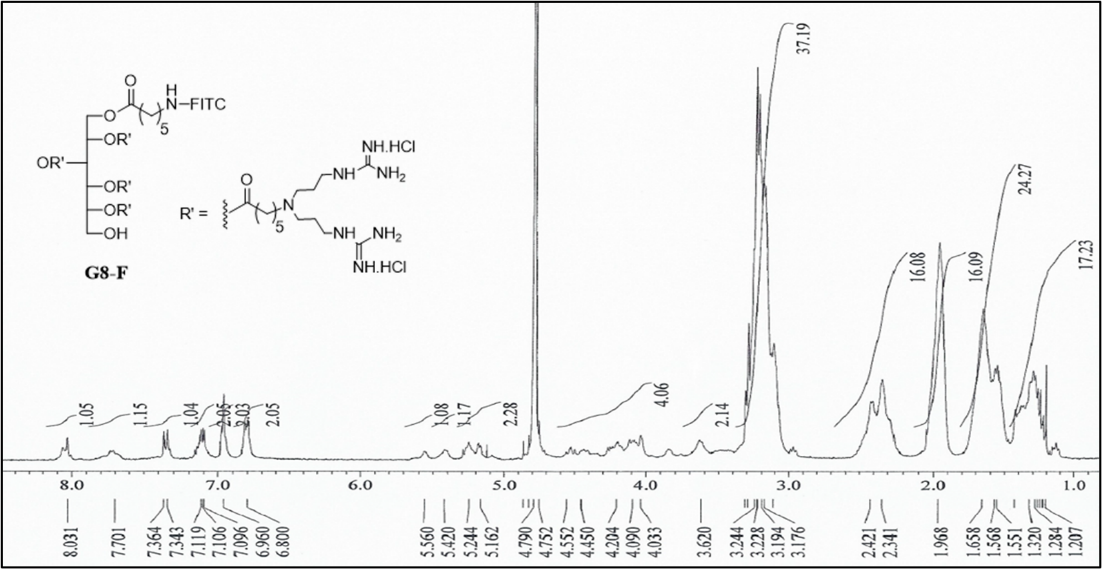
**(a)

**
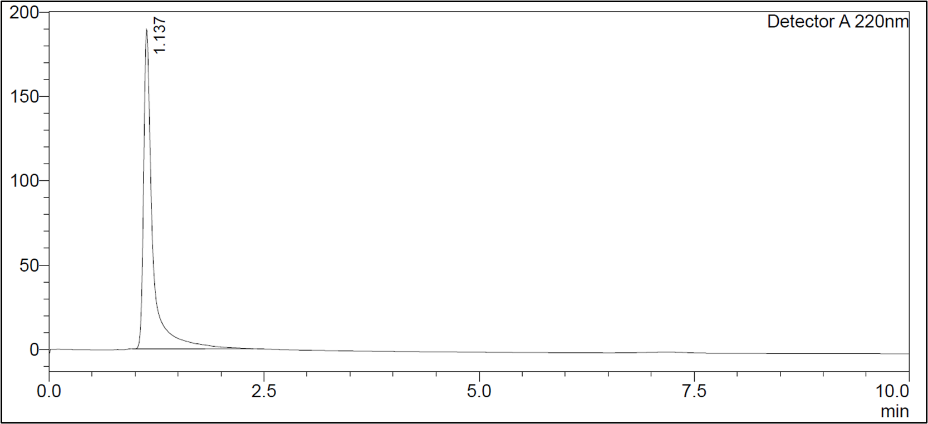
**(b)

**Figure S4.** Characteristic data of **G8-F**. (a) ^1^H-NMR spectrum, (b) RP-HPLC analysis: analytical HPLC (C18 monochromatic RP column, 220 nm, 1 mL/min, 30% ACN in H_2_O for 10 min, t_R_ = 1.13 min, showed the purity > 99%.

**3. Partition coefficients of the molecular transporters**


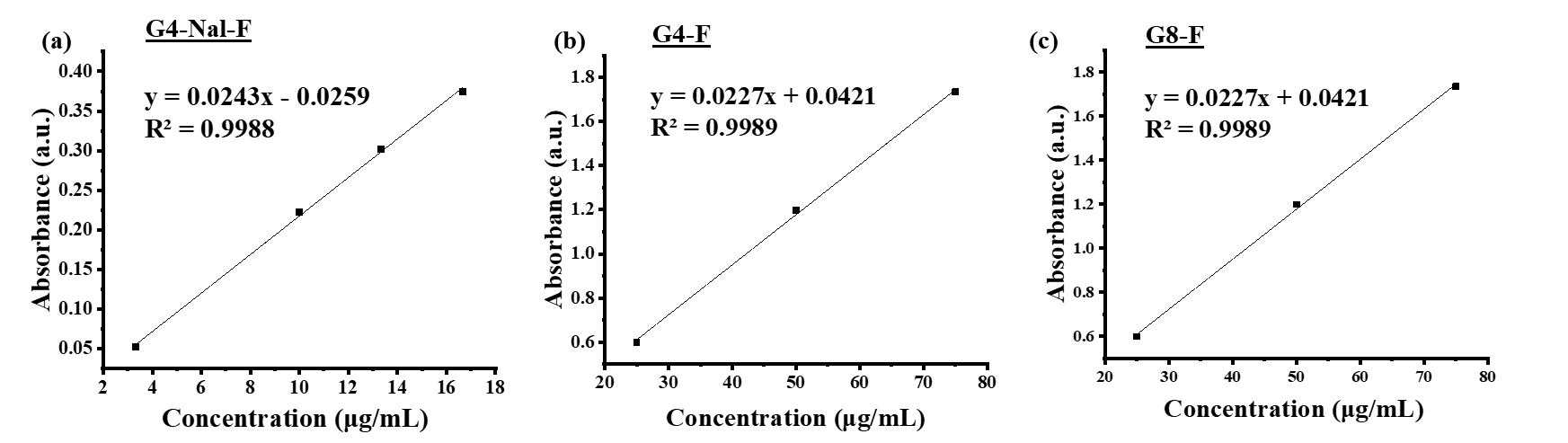


**Figure S5.** (a) Calibration curves of **G4-Nal-F**, (b) **G4-F**, and (c) **G8-F**. Each absorbance was measured by UV-Vis spectroscopy and the calibration curves were drawn against concentration (μg/mL) and absorbance.

Concentration of compounds distributed between aqueous and octanol layers after treating aqueous solution with octanol for 24 hr.

| Compound name | Absorbance | Conc.in H_2_O layer (ppm) | Conc.in Oct. layer (ppm) | Log P | |
| --- | --- | --- | --- | --- | --- |
| **G4-Nal-F** | 0.165 | 58.895 | 7.772 | | -0.879 |
| **G4-F** | 0.294 | 82.238 | 1.095 | | -1.876 |
| **G8-F** | 1.434 | 487.649 | 12.351 | | -1.596 |

**Table S1.** Determination of Log P values of respective compounds using absorbance and concentration, measured after treating aqueous layer with octanol for 24 hr.

**4. Cellular uptake mechanism study**


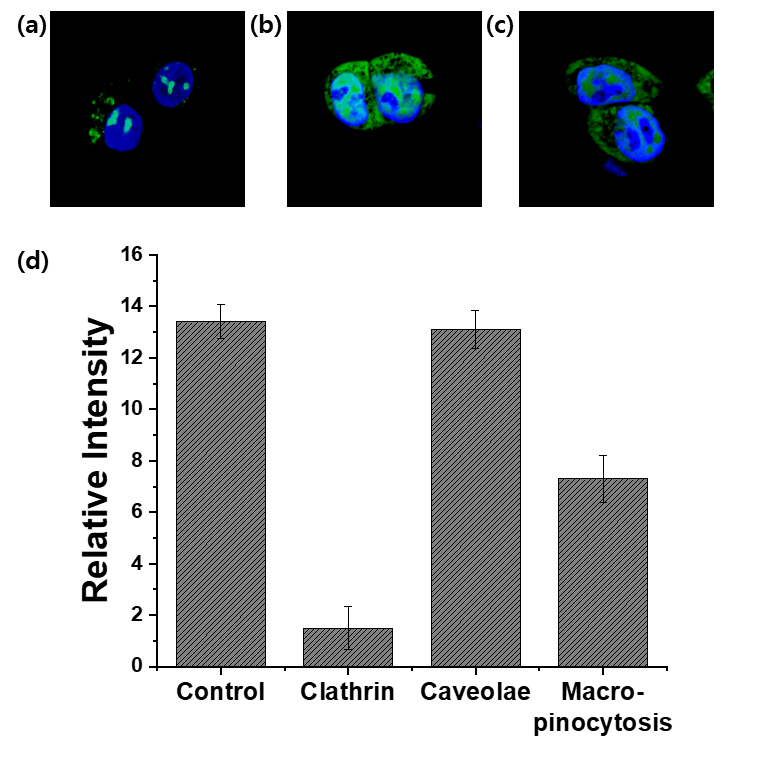


**Figure S6.** Study of cellular uptake mechanism of **G8-F** (2 μM) using endocytosis inhibitors. (a) clathrin-mediated endocytosis inhibition by chlorpromazine (30 μM), (b) caveolae-mediated endocytosis inhibition by methyl-b-cyclodextrin (10 mM), (c) macropinocytosis inhibition by imipramine hydrochloride (5 μM), (d) comparison of relative fluorescence intensity of **G8-F** under different inhibition conditions. **G8-F** incubation without an inhibitor is set as a control. The nucleus (blue color) was stained with Hoechst 33342 (Scale bar: 20 μm).

**5. Stability of G4-Nal, and IC**

***Sample Preparation***

The stability of **G4-Nal** was investigated in plasma and PBS at pH 7.4 using HPLC. First, **G4-Nal** was dissolved in H_2_O (100 μg/mL) to be a homogeneous solution. The solution was added to the plasma (plasma from human, Sigma, USA), filtered, and was made in a final concentration of 50 μg/mL for analysis. The sample was incubated at 37 °C, and aliquots (50 μL) were withdrawn at various time intervals (0, 0.5, 2.5, 3, 5, 15, and 21 hr), and were injected into the HPLC. The stability of **G4-Nal** in PBS was also investigated with the same analytical technique.

The stability of **IC** was investigated in aqueous with methanol using HPLC. First, **IC** was dissolved in MeOH (125 μg/mL) to be a homogeneous solution. The solution was added to 2 mL of H_2_O to make a clear solution, filtered, and was made in a final concentration of 62.5 μg/mL for analysis. Due to the low solubility of **IC** in aqueous solutions, **IC** was first dissolved in MeOH then added H_2_O to obtain a clear solution. The sample was incubated at 30 °C, and aliquots (100 μL) were withdrawn at various time intervals (0, 0.5, 1, 1.5, 2.5, 3, 5, 7.5, 10, 15, and 21 hr), and were injected into the HPLC.

***Data analysis***

The stability of **G4-Nal**, and **IC** were determined by the HPLC chromatograms. The mobile phases in HPLC were composed of MeOH (60%)/H_2_O in an isocratic mode with 1.0 mL/min flow rate for **G4-Nal**, and ACN (50%)/H_2_O in a gradient mode with 2.0 mL/min flow rate for **IC** to analyze the stability of **G4-Nal**, and **IC**, respectively. The UV detector was operated at 220 nm, and the column oven was maintained at 37 °C with an analytical column (shim-pack GIS C18, 4.6 x 150 mm, 5 μm). The percentage of the remaining amounts of **G4-Nal** and **IC** at each time interval was calculated from the peak height of initial concentrations of **G4-Nal** and **IC**. As shown in Fig. S7, after 30 min of incubation, **G4-Nal** was hydrolyzed about 0.58 % in PBS, and 0.15% in plasma with respect to the initial concentration. After 21 hr of incubation time, the percentage of concentration of **G4-Nal** reduced to 93.6% in PBS, and 81.3% in plasma (Fig. S7). This result indicates that **G4-Nal** has enough stability and long half-life in both plasma and PBS. **IC** was hydrolyzed about 0.44 % after 30 min of incubation with respect to the initial concentration. After 21 hr of incubation time, the percentage of concentration of **IC** was reduced to 86.6% (Fig. S8). The result from the stability test of **IC** indicates that **IC** has enough stability and long half-life after drug conjugation with the carrier molecule **G4-Nal**. Although **IC** was made by ionic interactions between charged molecules (**GA-Acid** and guanidiniums), the ionic interactions were stable at external conditions such as temperature and diverse solvents, showed high stability. This has shown that the drugs can be made by ionic interactions with great stability to avoid the extra work of synthesis.

**Figure S7.** The remained percentage of **G4-Nal** in PBS and human plasma at 37 °C. Aliquots were extracted at various time intervals and analyzed using HPLC.

**Figure S8.** The remained percentage of **IC** in 1:1 aqueous solution in MeOH at 30 °C. Aliquots were extracted at various time intervals and analyzed using HPLC.

**6. Cell viability test by MTT assay**

**Figure S9.** Cell viability of **G4-Nal**, **GA**, and **IC** in RAW 264.7 mouse macrophage cells (normal cells), based on the MTT assay. **GA**: geldanamycin, **IC**: ionic complex between **G4-Nal**/**GA-Acid** in (1:3) composition.
